# Salivary Proteomic Signatures Reveal Novel Mechanistic Insights into Moderate Dental Fluorosis Pathogenesis and Oral Homeostasis Disruption

**DOI:** 10.34133/csbj.0003

**Published:** 2026-03-16

**Authors:** Ansaya Pumchan, Patcharaporn Gavila, Thutchima Khieota, Han-Sung Jung, Kanokwan Sriwattanapong, Thantrira Porntaveetus

**Affiliations:** ^1^ Center of Excellence in Precision Medicine and Digital Health, Department of Physiology, Faculty of Dentistry, Chulalongkorn University, Bangkok 10330, Thailand.; ^2^ Intercountry Centre for Oral Health, Department of Health, Ministry of Public Health, Chiang Mai 50000, Thailand.; ^3^ Division in Anatomy and Developmental Biology, Department of Oral Biology, Taste Research Center, Oral Science Research Center, BK21 FOUR Project, Yonsei University College of Dentistry, Seoul, South Korea.; ^4^ Oral Biology Research Center, Faculty of Dentistry, Chulalongkorn University, Bangkok 10330, Thailand.; ^5^ Geriatric Dentistry and Special Patients Care International Program, Future Dent Digital Center, Faculty of Dentistry, Chulalongkorn University, Bangkok 10330, Thailand.

## Abstract

Moderate dental fluorosis (MF) represents a pivotal yet understudied stage of enamel fluorosis, characterized by distinct opacities indicating substantial but incomplete enamel disruption. Despite its prevalence, the molecular basis underlying this transitional phenotype remains unclear. This study employed a system proteomic approach to delineate salivary proteomic alterations associated with MF and to elucidate their biological and mechanistic implications. Unstimulated whole saliva was collected from school-aged children with MF (*n* = 10) and age-matched controls without fluorosis (CF, *n* = 21) for comparative proteomic analysis using liquid chromatography–tandem mass spectrometry. Urinary and drinking water fluoride levels were assessed to confirm comparable exposure between groups. Differentially expressed proteins (DEPs) were identified and subjected to Gene Ontology enrichment analysis, pathway analysis, and protein–protein interaction network analysis. Among 101 shared salivary proteins, 12 DEPs were significantly different between MF and CF subjects. Up-regulated proteins (e.g., neutrophil defensin 3, protein LEG1 homolog, immunoglobulin kappa constant, pyruvate kinase, mucin-7, and alpha-enolase) converged on pathways related to immune activation and altered glycolytic metabolism. Conversely, down-regulated proteins (e.g., superoxide dismutase [Cu-Zn], neutrophil gelatinase-associated lipocalin, thymidine phosphorylase, metalloproteinase inhibitor 1, galectin-3-binding protein, and alpha-1B-glycoprotein) reflected compromised antioxidant defense and perturbed extracellular matrix remodeling. Network topology analysis revealed distinct MF-specific interactomes dominated by immune–epithelial clusters. Notably, the cystic fibrosis transmembrane conductance regulator emerged as a central hub connecting multiple DEPs and MF-exclusive proteins, suggesting that fluoride-mediated ion transport perturbation may underlie enamel hypomineralization and broader oral homeostatic imbalance. Collectively, these findings provide novel molecular insights into MF pathogenesis and establish salivary proteomic profiles as a promising noninvasive platform for biomarker discovery, disease monitoring, and understanding of fluorosis progression.

## Graphical Abstract

**Figure:**
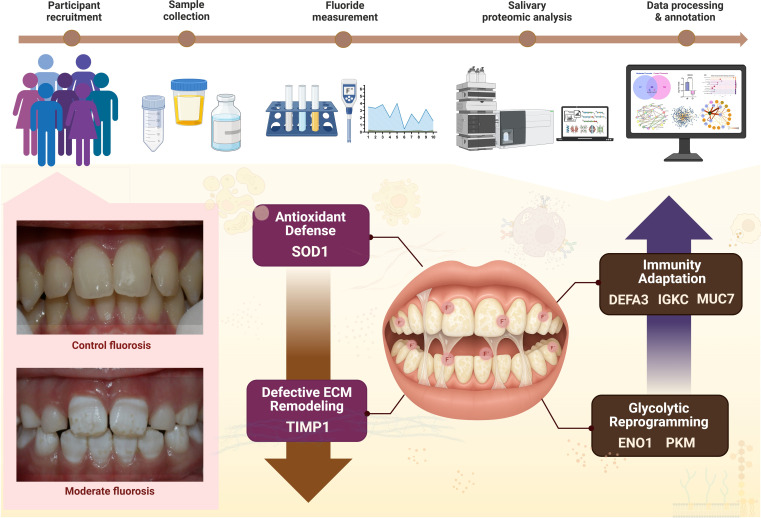

## Introduction

Fluoride is a trace element with well-documented cariostatic benefits when consumed at optimal levels, primarily through enhancing enamel remineralization and reducing bacterial acidogenicity [1]. However, when systemic exposure surpasses physiological thresholds, especially during the transition and maturation stages of enamel development, it can disrupt amelogenesis, resulting in dental fluorosis, a hypomineralization defect of enamel [2,3]. Clinically, this condition presents along a spectrum: mild forms show faint white opacities, moderate dental fluorosis (MF) displays more pronounced discoloration and porosity, and severe cases exhibit overt enamel breakdown, pitting, and brown staining [4,5].

Dental fluorosis, a pervasive global public health concern, spans a spectrum of enamel defects resulting from chronic fluoride overexposure during tooth development. While mild forms are often subtle and aesthetically innocuous and severe forms involve irreversible structural loss, MF holds a uniquely critical position as a biological transition state, poised between healthy enamel and advanced pathology. Clinically, MF is distinguished by both aesthetic and structural alterations; lesions with Thylstrup and Fejerskov (TF) index scores of 3 and 4 are already considered aesthetically compromising, exhibiting distinct white opacities and striations that may be accompanied by brownish discoloration [6]. This stage marks the point at which enamel opacity and early structural compromise become unambiguously evident. Importantly, the development of MF is often driven by aggregate fluoride exposure from multiple sources, including drinking water, food, discretionary salt, and ingested dental care products [7,8]. This cumulative daily intake can surpass physiological thresholds during the critical years of enamel formation, leading to the molecular and structural perturbations that define the onset of MF. Consequently, MF provides a crucial window in investigating the molecular tipping points and early pathogenic mechanisms before the disease progresses to more extensive and irreversible damage. Despite its prevalence and clinical relevance, the precise molecular mechanisms for distinguishing MF from milder forms and governing its progression toward severe pathology remain poorly defined.

Advances in high-throughput proteomics now allow the systematic characterization of saliva, transforming it into a powerful molecular window for disease research [9,10]. Saliva is a particularly attractive biofluid: it is noninvasive to collect, contains proteins secreted directly from salivary glands and gingival crevicular fluid, and reflects early immune and metabolic responses [11]. Importantly, altered salivary protein profiles have already been linked to several oral diseases, including dental caries, oral cancers, periodontitis, amelogenesis imperfecta, and molar–incisor hypomineralization, underscoring their utility in capturing disease-specific molecular signatures [12–14].

Investigating the salivary proteome in MF offers a unique opportunity to identify the early molecular signatures of fluoride toxicity. This approach promises to enhance our understanding of critical fluoride exposure thresholds and guide targeted strategies for prevention and early intervention, thereby mitigating the risk of irreversible enamel damage. Building on this rationale, our study focused on school-aged children from fluoride-endemic regions of Lamphun and Ratchaburi provinces, Thailand, where natural groundwater contamination poses a significant public health challenge. By employing comparative salivary proteomics, we aimed to uncover differentially expressed proteins and potential molecular biomarkers that reflect the specific biological responses to chronic fluoride exposure characteristic of MF. To robustly assess exposure, urinary fluoride concentrations were also quantified. This comprehensive approach was designed to provide novel molecular insights into the pathogenesis of dental fluorosis, bridging a critical knowledge gap between healthy enamel, moderate pathology, and severe disease stages.

## Materials and Methods

### Study design and participant recruitment

The cross-sectional study was conducted from December 2021 to January 2022 in fluoridated water regions of Lamphun and Ratchaburi provinces, Thailand. Participants, aged 6 to 16 years, were systematically recruited through collaboration with local schools and public health centers. Before enrollment, the study objectives, procedures, and potential benefits were described to children and their legal guardians during organized briefing sessions. Written informed consent from guardians and assent from the participants were subsequently obtained.

Participants were categorized into 2 groups based on their clinical dental fluorosis status: those with MF and those without fluorosis (control fluorosis [CF] group). Dental examinations were performed using the TF index, with scores of 3 and 4 designating the MF group and a score of 0 defining the CF group. Two calibrated evaluators independently performed all assessments, and a consensus diagnostic score was recorded on a standardized electronic form. The reliability of scoring was confirmed by calculating the intraclass correlation coefficient, which demonstrated excellent agreement: 0.989 for interobserver, 0.999 for intraobserver 1, and 1.000 for intraobserver 2. To ensure data validity, individuals were excluded if they presented with systemic disease, active caries, amelogenesis imperfecta, or any signs of oral mucosal lesions or periodontitis or if they had used antibiotics, antifungals, antivirals, corticosteroids, or mouthwash within the previous 3 months. Additional exclusion criteria include smoking, alcohol use, or narcotic use.

### Ethical considerations and sample collection

All study procedures adhered to the principles of the Declaration of Helsinki and received approval from the Human Research Ethics Committee of the Faculty of Dentistry, Chulalongkorn University, Thailand (HREC-DCU 2021-064; approval date: 2021 October 1). Prior to enrollment and sample collection, written informed consent was secured from all participating children and their legal guardians. Saliva and urine samples were collected within the participants’ schools under standardized conditions to certify sample integrity and comparability.

### Urine sample collection and fluoride measurement

Each participant provided a 24-h urine sample using 2.7-l polyethylene containers. The first morning void was discarded, and all subsequent urine in the next 24 h was collected into a wide-necked container and later transferred into screw-cap bottles. Samples were maintained at 4 °C during transport. Fluoride concentrations were measured using a fluoride-ion-selective electrode (Orion 4-Star Benchtop, Thermo Fisher, USA) in combination with Total Ionic Strength Adjustment Buffer III (TISAB III buffer) to stabilize pH and prevent ion interference. All analyses were conducted at the Intercountry Centre for Oral Health in Chiang Mai, Thailand.

### Drinking water collection and analysis

Participants were instructed to collect samples of their most frequently consumed household drinking water in clean, labeled 60-ml containers (filling to approximately five-sixths capacity). Samples were transported under controlled conditions to the Intercountry Centre for Oral Health, Chiang Mai, Thailand, where fluoride concentrations were determined using a fluoride-ion-selective electrode in combination with TISAB III, following the same protocol used for urine fluoride quantification.

### Saliva sample collection and processing

To minimize confounding variables, participants adhered to a strict regimen prior to specimen collection: an overnight fast of at least 12 h and abstinence from oral hygiene practices, food, and beverages on the morning of sampling. Unstimulated whole saliva was collected between 8:00 and 10:00 AM. Following a 30-s rinse with 10 ml of sterile water to remove oral debris and hydrate the mucosa, approximately 3 ml of saliva was passively drooled into sterile collection tubes. Samples were placed on ice immediately, rapidly transferred to dry ice for transport, and then stored long-term at −80 °C until proteomic analysis.

### Sample preparation and mass spectrometry analysis

Saliva samples were first clarified by centrifugation at 14,000 × g for 20 min at 4 °C to remove debris. Proteins in the supernatant were then precipitated with 100% trichloroacetic acid at a 1:10 ratio. After incubating on ice for 30 min, the protein pellet was collected by centrifugation at 20,000 × g for 20 min at 4 °C. The pellet was washed twice with 500 μl of pre-chilled acetone, with vortexing and a 10-min centrifugation at 20,000 × g after each wash. Finally, the washed pellet was resolubilized in 300 μl of 8 M urea in 100 mM triethylammonium bicarbonate containing a protease inhibitor cocktail (Halt, Thermo Fisher Scientific, USA).

For protein digestion, samples were reduced with dithiothreitol (10:1 ratio) at 37 °C for 30 min, followed by alkylation with 100 mM iodoacetamide for 30 min in the dark at room temperature. Excess iodoacetamide was quenched with 100 mM dithiothreitol for 15 min. The urea concentration was diluted to ˂ 1 M with 100 mM triethylammonium bicarbonate before adding trypsin (1:50 w/w; enzyme:substrate) for overnight digestion at 37 °C. The reaction was terminated with 0.5% trifluoroacetic acid (v/v) and incubated for 15 min at room temperature. Peptides were dried in SpeedVac, quantified using the Pierce Quantitative Fluorometric Peptide Assay, and labeled with Tandem Mass Tag (TMT10plex, Thermo Fisher Scientific, USA) according to the manufacturer’s instructions. Labeled peptides were fractionated using the High pH Reversed-Phase Peptide Fractionation Kit (Pierce), dried, and stored at −80 °C until analysis by mass spectrometry.

For liquid chromatography–tandem mass spectrometry (LC–MS/MS) analysis, peptides were reconstituted in 0.1% formic acid and separated on a nano-liquid chromatography system (EASY-nLC 1000) equipped with a Q Exactive Orbitrap Plus mass spectrometer (Thermo Fisher Scientific, USA). The separation was performed at a flow rate of 300 nl/min on a 25-cm C18 EASY-Spray column (75-μm inner diameter) over a 90-min linear gradient. Ionization was achieved via nano-electrospray at 2.0 kV. Full MS1 spectra were acquired from *m*/*z* 350 to 1,400 at a resolution of 70,000 (automatic gain control [AGC] target: 3 × 10^6^; maximum injection time: 250 ms). Data-dependent MS/MS acquisition targeted the 10 most abundant ions, fragmented by higher-energy collisional dissociation (NCE 32). MS2 spectra were recorded at a resolution of 35,000 (AGC target: 1 × 10^5^; maximum injection time: 100 ms).

### Proteomic data processing and functional annotation

Raw LC–MS/MS spectra were processed using Proteome Discoverer v2.1 (Thermo Fisher Scientific, USA) and matched to the Swiss-Prot human protein databases, supplemented with common contaminant proteins. Mass tolerance thresholds were set at 10 ppm for precursor ions and 0.02 Da for fragment ions. Only peptides and proteins identified with a false discovery rate <1% were retained. Reporter ion intensities for MF and CF groups were log_2_-transformed, and statistical analysis was performed using an unpaired *t* test on proteins with at least 3 valid quantitative values per group. Benjamini–Hochberg correction was applied, and proteins with a fold change >1.5 and an adjusted *P* <0.05 were considered significantly differentially expressed. Processed salivary proteomic data supporting this study have been deposited in the Zenodo repository (https://doi.org/10.5281/zenodo.18322088).

Functional annotation and enrichment analyses were performed using the Database for Annotation, Visualization, and Integrated Discovery (DAVID) Bioinformatics Resources (https://davidbioinformatics.nih.gov/tools.jsp) in conjunction with the Search Tool for the Retrieval of Interacting Genes (STRING) database (version 12.0; https://string-db.org). Gene Ontology (GO) enrichment analysis was conducted to classify proteins into the biological process (BP), molecular function (MFF), and cellular component (CC) categories. Pathway enrichment analysis was performed using the Kyoto Encyclopedia of Genes and Genomes (KEGG), Reactome, and WikiPathways databases. Pathway enrichment analysis was performed using an overrepresentation analysis framework, in which enrichment significance is assessed relative to the background annotation associated with each input protein list, rather than by direct pathway-level comparison between the MF and CF groups. The input for GO, pathway, protein–protein interaction (PPI), and network analyses consisted of proteins identified as differentially expressed between MF and CF (12 proteins), as well as proteins exclusively detected in the MF group in the proteomic analysis. PPI network analyses were performed using a 2-step workflow. First, PPI information was obtained from established interaction databases, including STRING, to capture known and predicted protein–protein associations. Second, molecular interaction networks were constructed and visualized in Cytoscape (version 3.10.3) using the IntAct app, which enables the generation of interaction networks based on curated molecular interaction evidence. This 2-step PPI analysis was used to explore known and predicted PPIs and to infer functional connectivity among proteins within the dataset. PPI network analysis was used as a systems-level, hypothesis-generating approach and does not represent direct measurements of protein abundance or differential expression; network centrality reflects inferred interaction relationships based on curated knowledge bases rather than experimental quantification.

### Statistical analysis

Demographic characteristics and fluoride concentrations in urine and water samples were summarized using descriptive statistics. Data normality was evaluated via the Shapiro–Wilk test. Comparisons of fluoride concentrations between the MF and CF groups were determined using the Mann–Whitney test. Differences in age and in up-regulated protein expression were analyzed with an unpaired *t* test with Welch’s correction. Sex distribution between groups was evaluated using the chi-square (Fisher’s exact) test. A 2-tailed *P* value <0.05 was considered indicative of statistical significance. All statistical analyses were conducted using GraphPad Prism v9.

## Results

### Participant characteristics and fluoride exposure

**Fig. 1.**
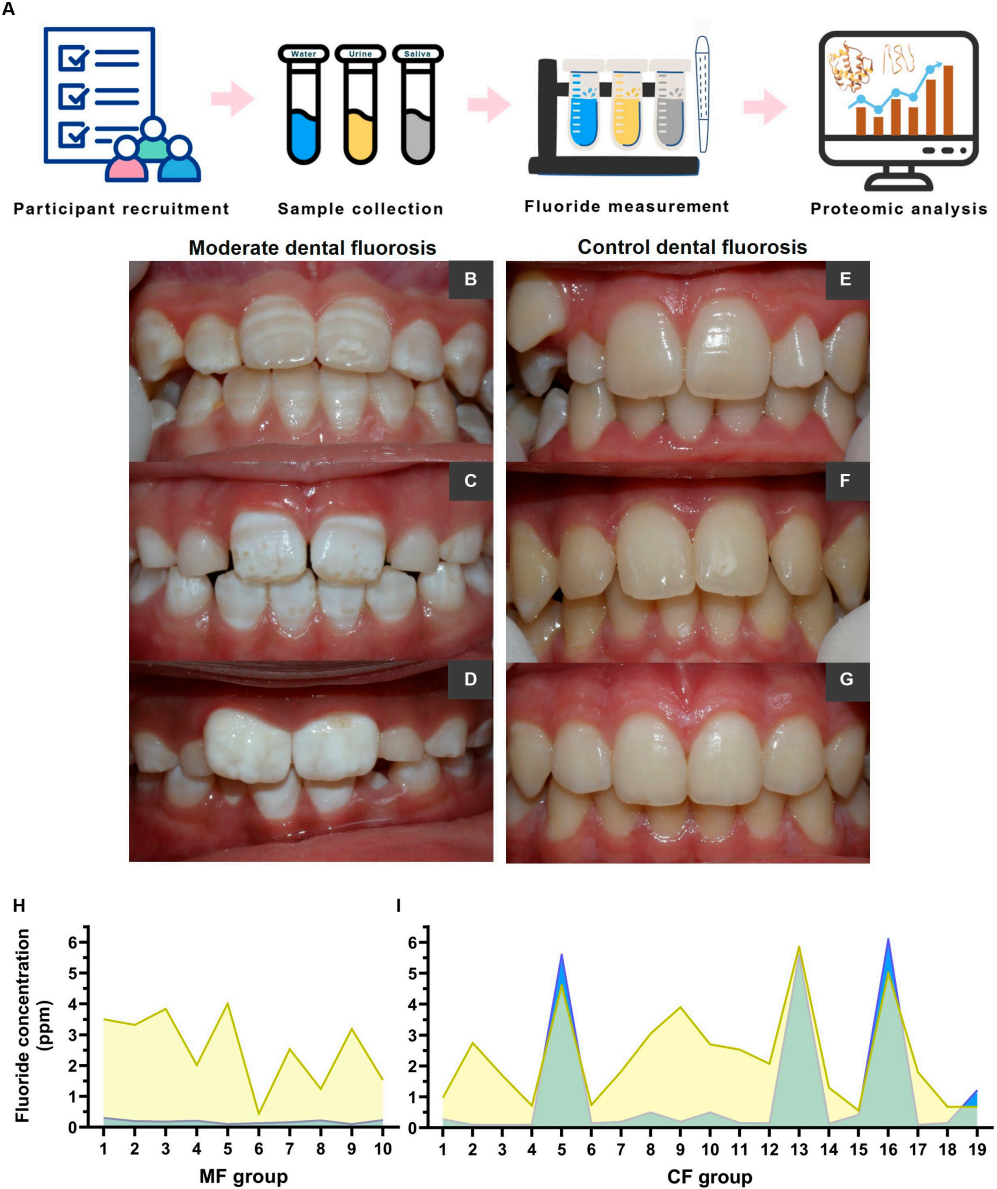
Dental characteristics of study participants and fluoride concentration in urine and drinking water. (A) Schematic summary of the study design. (B to D) Moderate dental fluorosis (MF) representing white opacities, yellow-brown speckles, surface irregularities, and pitting. (E to G) Control participants (control fluorosis [CF]) showing smooth, glossy, creamy-white enamel. (H and I) Fluoride concentrations (ppm) in urine (yellow) and water (blue) for individual participants in the MF (H) and CF (I) groups. Yellow plots indicate urinary fluoride levels, and blue overlays depict the corresponding water fluoride levels of each participant. (Note: Due to the technical limitations of data acquisition, the water fluoride concentration for participant MF06 was undetectable, as were both the water and urinary fluoride concentrations for participants CF20 and CF21.)

The comprehensive study workflow, encompassing participant recruitment, sample collection (drinking water, urine, and saliva), fluoride quantification, and salivary proteomic analysis, is summarized in Fig. 1A. Participants were stratified into 2 groups based on their TF fluorosis scores: the CF group (*n* = 21, TF score 0) and the MF group (*n* = 10, TF scores 3 and 4). Representative intraoral photographs of participants diagnosed in the MF group revealed enamel hypomineralization characterized by opaque white striations, mottled surface patterns, and diffuse opacities (Fig. 1B to D). Conversely, the CF group showed smooth, uniformly translucent enamel surfaces without visible defects (Fig. 1E to G).

Demographically, the participant ages were comparable between the CF (mean 10.74 ± 2.40 years) and MF (11.40 ± 0.97 years) groups (*P* = 0.302). However, a statistically significant difference was observed in sex distribution between the CF (male = 12; female = 9) and MF (male = 1; female = 9) groups (*P* = 0.02). Fluoride concentrations in drinking water and urine for each participant are illustrated in Fig. 1H and I. In the MF group, the mean urinary fluoride concentration was 2.56 ± 1.21 ppm (range: 0.43 to 4.01 ppm), while drinking water fluoride levels averaged 0.19 ± 0.06 ppm (range: <0.10 to 0.30 ppm). For the CF group, the mean urinary fluoride was 2.29 ± 1.60 ppm (range: 0.56 to 5.86 ppm), and water fluoride averaged 1.15 ± 2.07 ppm (range: <0.10 to 5.6 ppm). No statistically significant differences were detected between the groups for either urinary or drinking water fluoride concentrations. Notably, several CF participants exhibited transient elevations in urinary fluoride despite consistently low fluoride levels in their drinking water, suggesting the influence of additional or alternative exposure sources beyond the local water supply.

### Differential salivary proteomic signatures and functional enrichment in MF

**Fig. 2.**
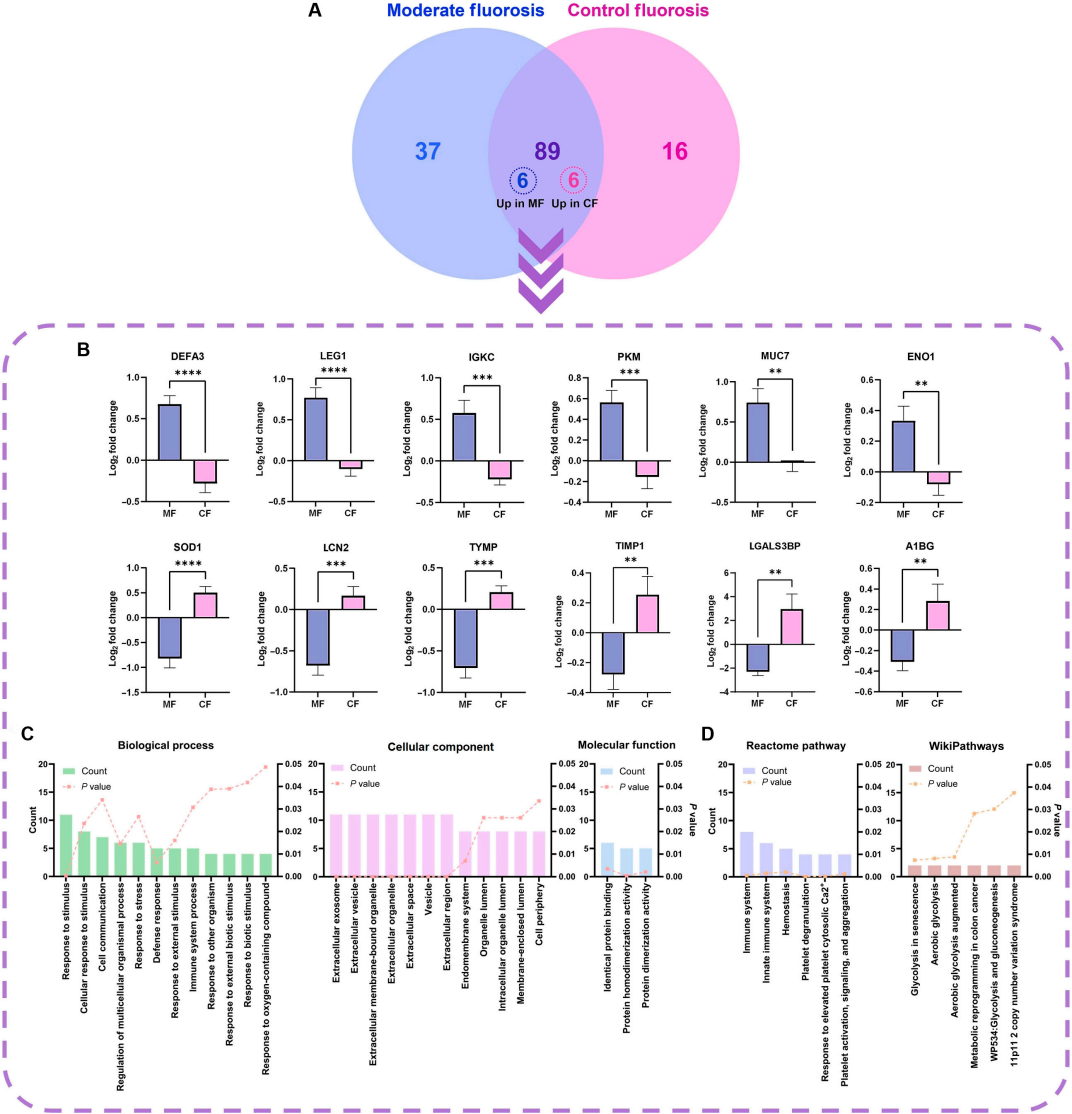
Salivary proteomic alterations in MF versus control (CF) participants. (A) Venn diagram showing the total of 154 proteins identified in the MF and CF groups, including 101 shared proteins and 6 proteins uniquely up-regulated in each group. (B) Log_2_ fold changes of significantly up-regulated salivary proteins in the MF (top) and CF (bottom) groups. Data depicted as mean ± standard error of the mean; statistical significance designated as *P* < 0.05 (*), *P* < 0.01 (**), *P* < 0.001 (***), and *P* < 0.0001 (****), and a ratio-fold change > 0.05. (C) Gene Ontology (GO) enrichment mapping of differentially expressed proteins, classified into biological processes (BP), cellular components (CC), and molecular functions (MFF). (D) Pathway enrichment characterization based on the Reactome and WikiPathways databases. For panels (C) and (D), bar graphs exhibit the number of proteins involved in each term (ranked by protein count); dashed lines denote statistical significance (*P* < 0.05). DEFA3, neutrophil defensin 3; LEG1, protein LEG1 homolog; IGKC, immunoglobulin kappa constant; PKM, pyruvate kinase; MUC7, mucin-7; ENO1, alpha-enolase; SOD1, superoxide dismutase [Cu-Zn]; LCN2, neutrophil gelatinase-associated lipocalin; TYMP, thymidine phosphorylase; TIMP1, metalloproteinase inhibitor 1; LGALS3BP, galectin-3-binding protein; A1BG, alpha-1B-glycoprotein.

Quantitative proteomic profiling identified a total of 166 proteins in saliva. After excluding contaminants and poorly labeled proteins, a high-confidence dataset of 161 proteins remained. To minimize the plausible bias arising from sporadic detection, proteins present in fewer than 3 samples per group were removed, resulting in a refined dataset of 154 proteins. Of these, 101 proteins were common to both groups, whereas 37 were uniquely detected in the MF group and 16 in the CF group (Fig. 2A).

Among the 101 shared proteins, 12 proteins exhibited statistically significant differences in abundance between groups: 6 were up-regulated and 6 were down-regulated in the MF group. The proteins found to be elevated in the MF group included neutrophil defensin 3 (DEFA3), protein LEG1 homolog (LEG1), immunoglobulin kappa constant (IGKC), pyruvate kinase (PKM), mucin-7 (MUC7), and alpha-enolase (ENO1). In contrast, the proteins with reduced abundance in the MF group were superoxide dismutase [Cu-Zn] (SOD1), neutrophil gelatinase-associated lipocalin (LCN2), thymidine phosphorylase (TYMP), metalloproteinase inhibitor 1 (TIMP1), galectin-3-binding protein (LGALS3BP), and alpha-1B-glycoprotein (A1BG) (Fig. 2B and Table S1). The key biological functions of these differentially expressed proteins, as annotated from the DAVID database, are listed in Table S1.

Functional enrichment analysis provided deeper insights into these proteomic shifts. GO terms for BP were enriched in functions related to inorganic and biotic stimuli, cell–cell communication, defense responses, and immune system processes. The most enriched CC terms included extracellular space, extracellular organelle, endomembrane system, and cell periphery. Meanwhile, MFF terms highlighted significant enrichment for protein binding and protein dimerization activity (*P* < 0.004) (Fig. 2C and Table S2). Pathway analysis, using Reactome and WikiPathways, further demonstrated significant enrichment in immune-system-related signaling, hemostasis, platelet degranulation, response to elevated platelet cytosolic Ca^2+^, glycolysis, and gluconeogenesis (Fig. 2D and Table S3). These findings suggest that the MF group is associated with distinct salivary proteomic signatures, reflecting not only the overt enamel pathology but also coordinated alterations in innate immune function, energy metabolism, and extracellular matrix (ECM) adaptations within the oral microenvironment.

### Functional characterization of group-exclusive salivary proteins

**Fig. 3.**
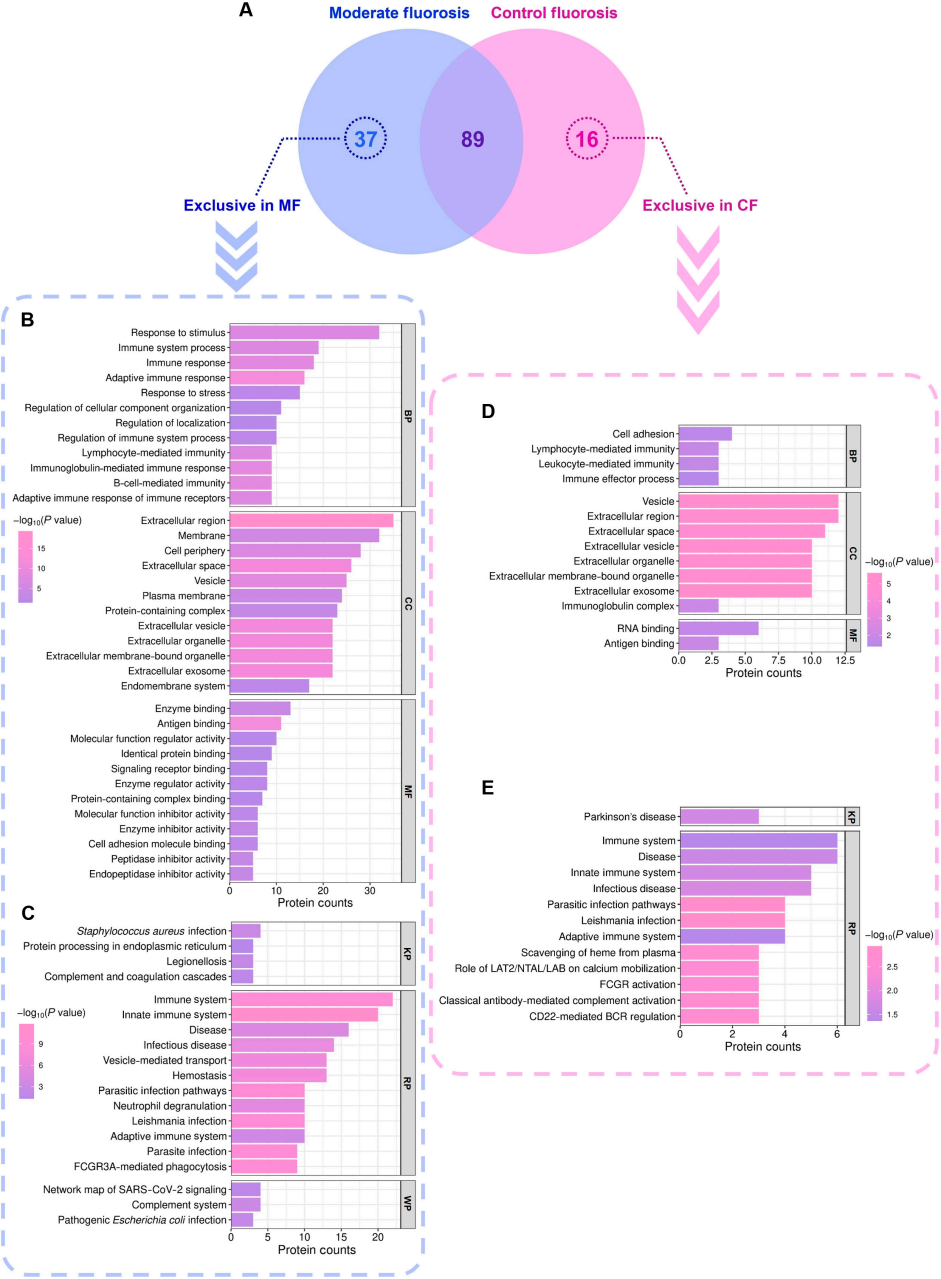
Functional and pathway enrichment analyses of proteins exclusively identified in the MF and CF groups. (A) Venn diagram depicting proteins uniquely detected in the MF group (37 proteins; purple) and the CF group (16 proteins; pink). (B and D) The GO enrichment analysis of MF-exclusive (B) and CF-exclusive (D) protein sets, categorized into biological process (BP), cellular component (CC), and molecular function (MF) terms. (C and E) Pathway enrichment analysis of MF-exclusive (C) and CF-exclusive (E) protein sets based on KEGG (KP), Reactome (RP), and WikiPathways (WP) databases. For all enrichment analyses, bar length represents the number of proteins associated with each term, and color intensity indicates statistical significance (*P* < 0.05).

Proteomic profiling revealed 37 proteins uniquely present in the MF cohort and 16 proteins exclusive to the CF group (Fig. 3A and Tables S4, S5, S8, and S9).

#### MF-exclusive proteins

GO enrichment of the MF-group-specific proteins demonstrated a strong overrepresentation of pathways involved in immune regulation and structural organization (Fig. 3B and C and Tables S6 and S7). The most significant enrichments within BP were for terms related to response to stimuli, innate and adaptive immune responses, and regulation of cellular component organization and localization. For the CC category, enrichment was dominated by terms associated with the extracellular region, followed by cell periphery, extracellular space, plasma membrane, extracellular vesicle/organelle, and endomembrane system. The MFF terms underscored that enzyme binding was the most dominant, with additional significant enrichment in antigen binding, enzyme regulator activity (specifically endopeptidase and peptidase), signaling receptor binding, cell adhesion molecule binding, and structural molecule activity (Fig. 3B and Table S6).

Pathway analyses confirmed these functional patterns, indicating distinct biological signatures in the MF group, particularly in immune- and defense-related pathways. Specifically, KEGG mapping indicated significant associations with *Staphylococcus aureus* infection, complement and coagulation cascades, and protein processing in the endoplasmic reticulum. Reactome further emphasized immune system functions, alongside additional immunological pathways. Consistently, WikiPathways enrichment identified the complement system as the most prominently represented pathway (Fig. 3C and Table S7).

#### Control-exclusive proteins

In contrast, the CF-exclusive proteins yielded fewer significant enrichments (Fig. 3D and E and Tables S10 and S11). BP enrichment was limited to cell adhesion, lymphocyte- and leukocyte-mediated immunity, and immune effector processes. CC enrichments largely pointed to extracellular-related compartments, while MFF terms included RNA binding and antigen binding (Fig. 3D and Table S10). Reactome pathway analysis highlighted immune-related processes such as parasitic infection, Fc gamma receptor activation, and calcium mobilization functions; however, these were detected at lower statistical significance compared with the highly enriched immune and extracellular pathways observed in the MF-exclusive proteins. This quantitative difference suggests that while both groups shared some involvement of immune-related processes, the magnitude and breadth of enrichment were far stronger in the MF group, consistent with more pronounced biological reprogramming under moderate fluoride exposure. In KEGG analysis, the only mapped pathway was Parkinson’s disease, while no significant enrichment was detected in WikiPathways (Fig. 3E and Table S11).

### PPI networks of differentially abundant and exclusive proteins

**Fig. 4.**
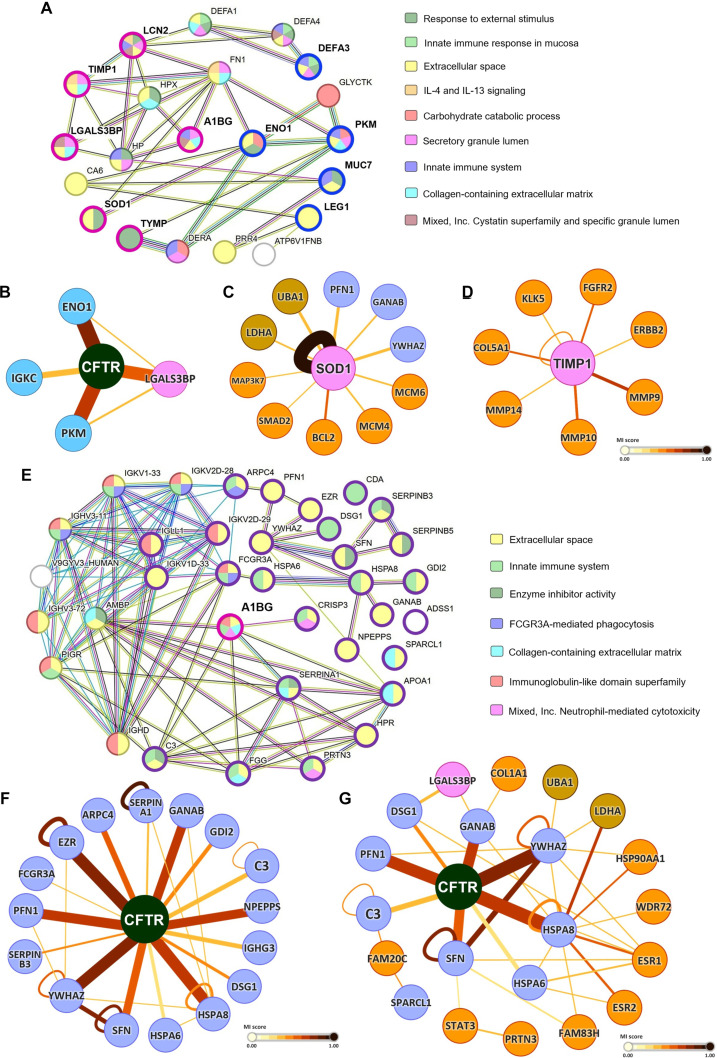
Integrative protein–protein interaction (PPI) and network mapping. (A and E) PPI networks generated using the Search Tool for the Retrieval of Interacting Genes (STRING) database based on (A) the 12 proteins differentially expressed between the MF and CF groups and (E) the 37 proteins exclusively detected in the MF group, illustrating known and predicted protein–protein associations derived from curated interaction databases. Proteins identified in the proteomic analysis are indicated as MF-up-regulated (blue), MF-down-regulated (pink), or MF-exclusive (purple) nodes. (B to D and F and G) Molecular interaction networks constructed and visualized in Cytoscape using the IntAct app (exact query mode) based on the same protein sets, enabling interaction-based network expansion and topological analysis. These Cytoscape-based networks illustrate inferred functional connectivity and potential clustering among the 12 differentially expressed proteins (B to D) and the 37 MF-exclusive proteins (F and G). Node shading denotes MF-up-regulated proteins (light blue), MF-down-regulated proteins (pink), MF-exclusive proteins (pastel purple), proteins previously associated with severe dental fluorosis (light brown), and proteins linked to other oral and dental diseases (orange). Edge confidence reflects interaction evidence scores. All networks represent inferred functional associations derived from curated interaction databases and do not reflect direct measurements of protein abundance or physical interactions. The molecular interaction (MI) score represents the confidence level of the interaction between 2 molecules based on available experimental and computational evidence.

To further elucidate the functional organization of differentially abundant salivary proteins, PPI networks were constructed and visualized in STRING and Cytoscape (Fig. 4).

#### PPI network of differentially abundant (shared) proteins

The network derived from the 12 shared proteins, which exhibited significant differential abundance between the MF and CF groups, revealed densely interconnected modules. These modules reflect coordinated activity across immune, metabolic, and extracellular functions (Fig. 4A). Specifically, half of the MF-down-regulated proteins clustered within the collagen-containing ECM (A1BG, TIMP1, and LGALS3BP) and the cystatin superfamily/specific granule lumen (A1BG, LGALS3BP, and LCN2). Furthermore, TIMP1 and LCN2 were directly linked to interleukin-4 and interleukin-13 signaling pathways, indicating their role in inflammatory processes. Conversely, the MF-up-regulated proteins, such as PKM and ENO1, were prominently localized within a carbohydrate metabolism module, whereas DEFA3 was positioned within an innate mucosal immune response cluster.

Crucially, network analysis highlighted central hub nodes, including CFTR, SOD1, and TIMP1, which served to bridge multiple immune, stress-response, ECM-mediation, and metabolic pathways (Fig. 4B to D). CFTR, in particular, demonstrated a critical integrating role, connecting the MF-up-regulated proteins (ENO1, PKM, and IGKC) with down-regulated proteins (LGALS3BP) (Fig. 4B). This suggests that CFTR acts as a pivotal integrator of fluoride-associated biological responses.

#### PPI network of group-exclusive proteins

For the 37 MF-exclusive proteins, the PPI analysis revealed a highly connected network organized into distinct clusters of immunoglobulin subunits, complement system proteins, and epithelial junctional components (Fig. 4E). Functional mapping showed strong enrichment for enzyme inhibitor activity, ECM proteins, and immune effectors involved in phagocytosis, complement activation, and adaptive immune signaling. Importantly, several MF-exclusive proteins (apolipoprotein A-I [APOA1], C3, isoform 3 of cysteine-rich secretory protein 3 [CRISP3], fibrinogen gamma chain [FGG], and alpha-1-antitrypsin [SERPINA1]) were directly connected to A1BG, a protein significantly down-regulated in the MF group. This direct link suggests a coordinated regulation between the loss of extracellular stability and compensatory immune activation.

Furthermore, CFTR emerged as a central bridging hub within the MF-exclusive protein network, directly linking 16 of these proteins. These interacting proteins spanned diverse functional categories, including structural proteins (desmoglein-1 [DSG1] and ezrin [EZR]), immune mediators (C3, immunoglobulin heavy constant gamma 3 [IGHG3], low affinity immunoglobulin gamma Fc region receptor III-A [FCGR3A], puromycin-sensitive aminopeptidase [NPEPPS], and SERPINA1), chaperones (isoform 2 of neutral alpha-glucosidase AB [GANAB], heat shock 70 kDa protein 6 [HSPA6], and heat shock cognate 71 kDa protein [HSPA8]), and key regulators of cell adhesion, migration, proliferation, and differentiation (isoform 3 of actin-related protein 2/3 complex subunit 4 [ARPC4], rab GDP dissociation inhibitor beta [GDI2], profilin-1 [PFN1], serpin B3 [SERPINB3], 14-3-3 protein sigma [SFN], and 14-3-3 protein zeta/delta [YWHAZ]) (Fig. 4F). The integrative role of CFTR was further emphasized by its interconnection with LGALS3BP, a significantly down-regulated protein in the MF group, which was also linked to 2 MF-exclusive proteins connected to CFTR (Fig. 4G). This underscores CFTR’s profound role as a key integrator of epithelial barrier function, immune adaptation, and broader oral homeostasis in the MF condition.

## Discussion

Dental fluorosis results from cumulative fluoride exposure through water, food, and oral care products, disrupting amelogenesis and leading to MF [7]. As a transitional stage marking key molecular and structural changes in enamel, MF provides a window into disease progression. This study identified salivary proteomic signatures that not only mirror enamel mineralization defects but also reveal systemic alterations in immunity, metabolism, and ECM regulation, offering potential biomarkers for early detection and prevention.

Interestingly, we observed comparable urinary and drinking water fluoride levels between our MF and control groups, echoing previous reports that conventional fluoride biomarkers do not always correlate directly with clinical severity [15]. This highlights that the biological impact of fluoride extends beyond a single-point concentration measurement, depending instead on cumulative intake from multiple sources and the host’s intrinsic ability to regulate fluoride metabolism [16]. Urinary fluoride was used as a biomarker of systemic exposure, reflecting cumulative fluoride exposure inferred rather than comprehensively quantified across all potential sources. Our findings thus reinforce the concept that salivary proteomics may provide a more dynamic and functionally relevant readout of biological responses to fluoride exposure than traditional methods.

Among the 12 significantly altered proteins, the up-regulation of DEFA3, MUC7, IGKC, LEG1, PKM, and ENO1 in the MF group collectively points to heightened immune activity and metabolic reprogramming. DEFA3, an antimicrobial peptide derived from neutrophils, suggests a compensatory defense mechanism aimed at protecting structurally compromised enamel from microbial adhesion and colonization [17]. The elevation of MUC7, being a major salivary mucin, reflects altered mucin composition, potentially contributing to chemical and antimicrobial defense [18]. Similarly, the increased abundance of IGKC indicates activation of adaptive immune pathways, consistent with its previously reported association with immune dysregulation in oral malignant disorders [19]. LEG1 was also elevated in the MF group. Although its role in oral biology has not been extensively characterized, LEG1 is a secreted protein expressed in epithelial and secretory tissues and has been implicated in mucosal defense and innate immune regulation [20,21]. Its detectability in saliva is therefore biologically plausible, and its increased abundance in MF may reflect broader mucosal or immune adaptations to fluoride-associated environmental stress rather than a fluorosis-specific effect. Metabolically, the rise in PKM and ENO1 underscores glycolytic reprogramming under fluoride stress. PKM, a pivotal regulator of glycolytic metabolism, influences immune cell infiltration, inflammation, and tissue remodeling, all of which are implicated in periodontal and neoplastic conditions [22,23]. The elevated ENO1 abundance may reflect a compensatory mechanism in response to fluoride’s competitive inhibition of ENO1, which can disrupt glycolytic flux and induce inflammatory stress [24,25]. Together, these findings align with experimental evidence that the up-regulation of glycolytic proteins can alter energy metabolism in ameloblasts and exacerbate fluorosis development in animal models [26]. Thus, the salivary proteome of the MF group reveals a pattern of immune priming and metabolic adjustment, reflecting both local enamel vulnerability and systemic biological responses to cumulative fluoride exposure.

In contrast to the up-regulated immune and metabolic proteins, a core feature of the MF group was the down-regulation of antioxidant and ECM-regulating proteins in saliva. The notable reduction of SOD1, an essential antioxidant enzyme expressed in the periodontal ligament and gingival fibroblasts, indicates a diminished capacity to neutralize superoxide anions [27]. Such depletion may heighten susceptibility to reactive oxygen species, disrupt mitochondrial redox homeostasis, and impair cell function [28], thereby exacerbating enamel pathology through compromised ameloblast activity [29]. Cytoscape-based PPI analysis further revealed SOD1 clustering with several MF-unique proteins, including GANAB, PFN1, and YWHAZ, which are associated with protein folding [30,31], cell migration [32,33], and cell proliferation and apoptosis [34,35]. Interestingly, SOD1 also showed marked interactions with several key mediators of tooth development, including mitogen-activated protein kinase (MAPK) signaling mediators (MAP3K7 and SMAD2), the anti-apoptotic regulator (BCL2 apoptosis regulator [BCL2]), and cell proliferation markers (minichromosome maintenance complex component 4 [MCM4] and minichromosome maintenance complex component 6 [MCM6]) [36,37]. This network also encompassed ubiquitin-like modifier-activating enzyme (UBA1; a ubiquitination enzyme) and isoform 3 of L-lactate dehydrogenase A chain (LDHA; a glycolytic enzyme), both previously reported to be up-regulated in severe fluorosis [10], suggesting that redox imbalance and metabolic adaptation in the MF group may converge with proteostasis and cell survival pathways that become more pronounced in advanced stages of the disease.

Similarly, the marked reduction in TIMP1, a key inhibitor of matrix metalloproteinases (MMPs), points to disturbed ECM turnover. Diminished TIMP1 may tip the balance toward unchecked MMP activity, which could weaken enamel-supporting structures and compromise tissue stability [38]. Network mapping linked TIMP1 to ECM remodeling proteins (MMP9, MMP10, MMP14, and collagen type V alpha 1 chain [COL5A1] [39]), as well as to growth factor receptors (fibroblast growth factor receptor 2 [FGFR2] [40] and Erb-B2 receptor tyrosine kinase 2 [ERBB2] [41]) and the cell–cell aggregation and cohesion mediator (kallikrein related peptidase 5 [KLK5] [42,43]). These associations implicate TIMP1 in broader processes of ECM remodeling, growth factor signaling, and cell–cell adhesion, all of which are highly vulnerable to fluoride-induced dysregulation in the oral environment.

The PPI network of several proteins exclusive to the MF cohort revealed reinforced interaction profiles, which may be attributable to chronic fluoride exposure. Beyond their associations with the CFTR channel, proteins such as YWHAZ and HSPA8 were notably linked to proteins previously documented as up-regulated in severe dental fluorosis [10]. This observation is further supported by the network’s inclusion of other proteins vital for tooth development and maintenance of oral health [44]. These included the stress-inducible chaperone heat shock protein 90 alpha family class A member 1 (HSP90AA1) [45], which is crucial for cell proteostasis, alongside key players in mineralization and ameloblast differentiation such as collagen type I alpha 1 chain (COL1A1) [46], estrogen receptor 1 (ESR1), estrogen receptor 2 (ESR2) [47], family with sequence similarity 20 C (FAM20C) [48], family with sequence similarity 83 member H (FAM83H) [49], signal transducers and activators of transcription 3 (STAT3) [50], and tryptophan-aspartate repeat domain 72 (WDR72) [51]. Notably, MF-exclusive salivary proteins converged within interaction networks centered on FAM20C, FAM83H, and COL1A1, which themselves interface with key enamel- and matrix-associated regulators, including family with sequence similarity 20, member A (FAM20A); enamelin (ENAM); and integrin beta-6 (ITGB6), implicated in enamel mineralization, ameloblast-related pathways, and ECM organization [52,53]. FAM20C, FAM20A, and FAM83H are involved in enamel matrix phosphorylation and maturation, whereas COL1A1 supports the structural integrity of mineralized tissues [54]. Given that the study cohort comprised children aged 6 to 16 years, encompassing developmental stages during which enamel formation of different tooth types may be ongoing or already complete, these salivary proteomic signatures are not interpreted as markers of active amelogenesis. Instead, the integration of enamel- and matrix-associated regulators within the MF-related PPI network suggests that chronic fluoride exposure may induce persistent molecular imprints linked to disrupted enamel biology, which remain detectable in saliva across developmental stages. Accordingly, salivary proteomics is employed as a noninvasive, hypothesis-generating approach, with careful consideration of its temporal and biological scope. These signals likely reflect a combination of enduring regulatory adaptations arising from earlier developmental exposure and secondary responses associated with altered enamel structure and its ongoing interaction with the oral environment, supporting the use of saliva as a noninvasive molecular window into fluorosis-associated biology [55,56]. Collectively, the dense functional interconnections within the MF-exclusive proteome underscore coordinated biological reprogramming processes that span oxidative stress, ECM integrity, and ameloblast function, distinguishing MF from mild forms and hinting at a trajectory toward more advanced pathology.

Network analysis provided further insight by revealing CFTR as a prominent bridging node connecting multiple functional modules within the inferred PPI network. Importantly, CFTR expression is highly cell type specific and temporally regulated, particularly during developmental processes such as amelogenesis [57,58]. Previous studies have shown that CFTR activity is enriched during discrete stages of enamel formation, most notably in maturation-stage ameloblasts, rather than being constitutively or continuously expressed in extracellular secretions such as saliva [59]. Accordingly, the absence of CFTR as a directly detected salivary protein in the present proteomic dataset does not preclude its functional or regulatory relevance within fluoride-associated molecular networks. Network analysis provided additional insight by identifying CFTR as a prominent bridging node connecting multiple functional modules within the inferred PPI network. Although CFTR was not experimentally detected as a differentially expressed salivary protein, its apparent centrality reflects inferred functional connectivity based on curated interaction databases. Within this network, CFTR was linked to proteins involved in glucose metabolism, immune modulation, protein folding, structural organization, protease regulation, and key cellular processes such as homeostasis, adhesion–migration, and proliferation–differentiation [19,24,25,30–35]. Together, these network-based associations highlight a context-dependent, integrative role for CFTR in coordinating fluoride-associated biological responses, potentially through developmentally restricted ion transport and pH-regulatory mechanisms rather than via direct salivary expression.

This interpretation is supported by prior studies reporting functional correlations between CFTR and up-regulated salivary proteins in severe dental fluorosis (histone H4 [H4C3], plastin-2 [LCP1], LDHA, S100 calcium binding protein A9 [S100A9], and UBA1), underscoring its possible involvement in immune, metabolic, and survival pathways under fluoride stress [10]. Moreover, urinary proteomic studies in children with high fluoride exposure have identified systemic alterations in ECM, biomineralization, oxidative stress, and immune pathways, with several proteins linked to CFTR. The emergence of CFTR as a network-inferred integrative node suggests that CFTR-mediated ion transport and pH regulation may represent a shared molecular axis linking systemic fluoride exposure to localized salivary and oral responses across different exposure severities [60]. Mechanistically, CFTR is a gated anion channel localized not only at the apical plasma membrane of epithelial cells, including ameloblasts, but also within intracellular organelles such as mitochondria and lysosomes [61]. Beyond its canonical role in chloride transport, CFTR orchestrates epithelial and intracellular pH regulation and ion homeostasis by mediating the exchange of glutathione, halides, and polyatomic anions [61]. Disruption of CFTR function has been shown to impair secretory processes and intracellular signaling, particularly pathways related to chemokine production and glucose metabolism [62,63], and has been consistently linked to incomplete enamel mineralization across porcine, rat, mouse, and human models [57].

Since fluoride can be transported through CFTR, excessive exposure may competitively interfere with anion exchange, especially during the maturation stage of amelogenesis, thereby potentially compromising enamel deposition and contributing to enamel hypomineralization [57]. Taken together, the CFTR-centered interaction patterns observed in MF may reflect an early stage of molecular dysregulation that parallels, and may precede, the proteomic disturbances documented in severe disease. In this context, MF could represent a transitional state in which emerging CFTR-linked alterations foreshadow the broader immune, metabolic, and structural disruptions characteristic of advanced pathology.

Although a statistically significant imbalance in sex distribution was observed between the CF and MF groups, available epidemiological evidence indicates that biological sex is not a dominant determinant of dental fluorosis prevalence or severity in pediatric populations [64–66]. While biological sex may influence certain immune and salivary parameters, enamel formation and amelogenesis during childhood are governed by conserved developmental processes that are not regarded as strongly sex dependent [67]. Accordingly, sex was not incorporated as a covariate in the proteomic analysis to avoid overparameterization in a high-dimensional dataset. This imbalance is therefore acknowledged as a methodological consideration, and future studies employing sex-balanced cohorts will be important to further delineate potential sex-specific contributions to fluoride-associated salivary proteomic alterations.

While strict inclusion criteria and standardized sampling procedures were applied to reduce biological and technical variability, this study was inherently exploratory and hypothesis generating, and the cohort size was necessarily limited. In addition, cumulative fluoride exposure was inferred rather than comprehensively quantified across all potential sources, with urinary fluoride serving as a biomarker of systemic exposure. Accordingly, the identified proteomic alterations should be interpreted as preliminary molecular signals associated with MF, reflecting potential inter-individual variability in biological responses, and warrant validation in larger, independent cohorts [60,68]. Importantly, this study establishes a foundational proteomic framework for MF while opening promising avenues for future research. Beyond protein abundance, incorporating posttranslational modifications and metabolomic data could uncover additional fluoride-related mechanisms. Longitudinal studies are crucial to clarify how these molecular patterns evolve during enamel maturation, and broader exposure assessments, integrating dietary and genetic factors, could refine susceptibility profiles. Together, these approaches would strengthen the translational potential of salivary proteomics as a noninvasive tool for early diagnosis and targeted prevention of fluorosis.

**Fig. 5.**
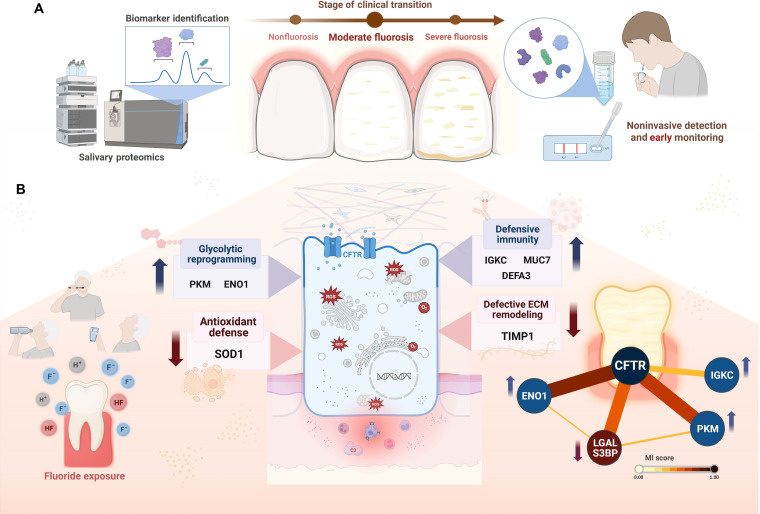
Summary schematic demonstrating the key findings of the study. (A) Clinical relevance: The findings suggest biological transition between clinical stages. Salivary proteomics offers a noninvasive platform for early detection and monitoring of fluorosis progression in response to cumulative fluoride exposure. ROS, reactive oxygen species.(B) Molecular signature: Salivary proteomics revealed a distinct profile in moderate fluorosis, by the up-regulation of immune (DEFA3, MUC7, and IGKC) and metabolic (PKM and ENO1) proteins and down-regulation of antioxidant (SOD1) and extracellular-matrix (ECM)-related (TIMP1) proteins. The cystic fibrosis transmembrane conductance regulator (CFTR) acted as a central hub coordinating these changes.

In conclusion, this study demonstrates that MF represents a biologically distinct stage marked by coordinated alterations in immunity, metabolism, antioxidant defense, and ECM regulation (Fig. 5). The up-regulation of DEFA3, MUC7, IGKC, PKM, and ENO1 suggests intensified defense and glycolytic reprogramming, while the down-regulation of SOD1 and TIMP1 reflects increased susceptibility to oxidative stress and defective ECM remodeling. CFTR emerged as a network-inferred central hub linking immune, metabolic, and structural pathways, consistent with its role in enamel mineralization and ion homeostasis. These proteomic shifts, observed despite similar urinary and water fluoride levels between groups, highlight the role of cumulative fluoride exposure from multiple sources. Importantly, the molecular alterations identified in MF may foreshadow the transition toward severe disease, underscoring the value of salivary proteomics as a noninvasive tool for early detection, monitoring, and prevention of this prevalent condition.

## Ethical Approval

Written informed consent was obtained from all participants involved in the study.

## Data Availability

The datasets generated and/or analyzed during this study are available from the corresponding author upon reasonable request.
