# Supplementary material for: Salivary Proteomic Signatures Reveal Novel Mechanistic Insights into Moderate Dental Fluorosis Pathogenesis and Oral Homeostasis Disruption
Source: Comput Struct Biotechnol J. 2026 Mar 16;35(1):0003. doi: 10.34133/csbj.0003 (PMC13068020; doi:10.34133/csbj.0003)
Supplement: Supplementary 1 — Tables S1 to S11 [file csbj.0003.f1.docx]

**Supplementary Table 1.** **Key biological functions of 12 proteins significantly altered in the MF group compared with the CF group.**

| **UniProtKB Entry** | **Gene** | **Protein name** | **Key biological function** |
| --- | --- | --- | --- |
| P59666 | DEFA3 | Neutrophil defensin alpha 3 | Innate immune response in mucosa, antibacterial humoral response, estrogen receptor signaling pathway, defense response to bacterium, fungus, and virus, antimicrobial humoral immune response mediated by antimicrobial peptide, cellular response to lipopolysaccharide |
| Q6P5S2 | LEG1 (C6orf58) | Protein LEG1 homolog | Uncategorized biological process function (based on DAVID analysis) |
| P01834 | IGKC | Immunoglobulin kappa constant | Adaptive immune response, immune response, immunoglobulin mediated immune response, B cell receptor signaling pathway |
| P14618 | PKM | Pyruvate kinase | Glycolytic process, chromatin remodeling, programmed cell death, cellular response to insulin stimulus, positive regulation of transcription by RNA polymerase II, positive regulation of cytoplasmic translation |
| Q8TAX7 | MUC7 | Mucin-7 | Killing of cells of another organism, antimicrobial humoral immune response mediated by antimicrobial peptide |
| P06733 | ENO1 | Alpha-enolase | Negative regulation of transcription by RNA polymerase II, gluconeogenesis, glycolytic process, response to virus, positive regulation of plasminogen activation, negative regulation of cell growth, negative regulation of DNA-templated transcription, negative regulation of hypoxia-induced intrinsic apoptotic signaling pathway, positive regulation of ATP biosynthetic process |
| P00441 | SOD1 | Superoxide dismutase [Cu-Zn] | Response to superoxide, positive regulation of cytokine production, glutathione and superoxide metabolic process, intracellular iron ion homeostasis, apoptotic process, gene expression, removal of superoxide radicals, regulation of T cell differentiation in thymus, regulation of GTPase activity, positive regulation of MAPK cascade, positive regulation of phagocytosis, positive regulation of oxidative stress-induced intrinsic apoptotic signaling pathway |
| P80188 | LCN2 | Neutrophil gelatinase-associated lipocalin | Apoptotic process, siderophore transport, defense response to bacterium, innate immune response, positive regulation of cold-induced thermogenesis |
| P19971 | TYMP | Thymidine phosphorylase | Mitochondrial genome maintenance, angiogenesis, pyrimidine nucleobase metabolic process, pyrimidine nucleoside metabolic process, chemotaxis, signal transduction, cell differentiation, regulation of myelination, dTMP catabolic process, regulation of transmission of nerve impulse, regulation of gastric motility |
| P01033 | TIMP1 | Metalloproteinase inhibitor 1 | Connective tissue replacement involved in inflammatory response wound healing, signal transduction, positive regulation of cell population proliferation, response to hormone, negative regulation of endopeptidase activity, response to cytokine, negative regulation of apoptotic process, negative regulation of catalytic activity, response to peptide hormone, negative regulation of membrane protein ectodomain proteolysis, cartilage development, cellular response to UV-A, negative regulation of trophoblast cell migration, cellular response to peptide, negative regulation of metallopeptidase activity, regulation of integrin-mediated signaling pathway, |
| Q08380 | LGALS3BP | Galectin-3-binding protein | Cellular defense response, cell adhesion, signal transduction, vesicle-mediated transport |
| P04217 | A1BG | Alpha-1B-glycoprotein | Immune response-regulating signaling pathway |

**Supplementary Table 2.** **Gene Ontology profiles of 12 upregulated genes in the MF and CF groups.**

| **Category/Term** | **Count** | **Genes** | ***p*-value** | **List total** | **Pop hits** | **Pop total** | **Fold enrichment** | **Bonferroni** | **Benjamini** | **FDR** |
| --- | --- | --- | --- | --- | --- | --- | --- | --- | --- | --- |
| **Biological process** |  |  |  |  |  |  |  |  |  |  |
| GO:0050896~response to stimulus | 11 | LGALS3BP, PKM, IGKC, MUC7, LCN2, DEFA3, A1BG, TIMP1, ENO1, TYMP, SOD1 | 2.84E-04 | 11 | 8582 | 19416 | 2.2624 | 0.1675 | 0.1833 | 0.1833 |
| GO:0051716~cellular response to stimulus | 8 | LGALS3BP, PKM, IGKC, DEFA3, A1BG, TIMP1, TYMP, SOD1 | 0.0245 | 11 | 6679 | 19416 | 2.1142 | 1.0000 | 0.6484 | 0.6484 |
| GO:0007154~cell communication | 7 | LGALS3BP, IGKC, DEFA3, A1BG, TIMP1, TYMP, SOD1 | 0.0341 | 11 | 5433 | 19416 | 2.2742 | 1.0000 | 0.6484 | 0.6484 |
| GO:0051239~regulation of multicellular organismal process | 6 | PKM, LCN2, TIMP1, ENO1, TYMP, SOD1 | 0.0146 | 11 | 3194 | 19416 | 3.3158 | 0.9999 | 0.6484 | 0.6484 |
| GO:0006950~response to stress | 6 | LGALS3BP, MUC7, LCN2, DEFA3, TIMP1, SOD1 | 0.0266 | 11 | 3689 | 19416 | 2.8708 | 1.0000 | 0.6484 | 0.6484 |
| GO:0006952~defense response | 5 | LGALS3BP, MUC7, LCN2, DEFA3, TIMP1 | 0.0062 | 11 | 1589 | 19416 | 5.5541 | 0.9829 | 0.6484 | 0.6484 |
| GO:0009605~response to external stimulus | 5 | MUC7, LCN2, DEFA3, ENO1, TYMP | 0.0160 | 11 | 2072 | 19416 | 4.2594 | 1.0000 | 0.6484 | 0.6484 |
| GO:0002376~immune system process | 5 | IGKC, MUC7, LCN2, DEFA3, SOD1 | 0.0307 | 11 | 2511 | 19416 | 3.5147 | 1.0000 | 0.6484 | 0.6484 |
| GO:0051707~response to other organism | 4 | MUC7, LCN2, DEFA3, ENO1 | 0.0388 | 11 | 1535 | 19416 | 4.5996 | 1.0000 | 0.6484 | 0.6484 |
| GO:0043207~response to external biotic stimulus | 4 | MUC7, LCN2, DEFA3, ENO1 | 0.0390 | 11 | 1538 | 19416 | 4.5906 | 1.0000 | 0.6484 | 0.6484 |
| GO:0009607~response to biotic stimulus | 4 | MUC7, LCN2, DEFA3, ENO1 | 0.0418 | 11 | 1580 | 19416 | 4.4686 | 1.0000 | 0.6590 | 0.6590 |
| GO:1901700~response to oxygen-containing compound | 4 | PKM, DEFA3, TIMP1, SOD1 | 0.0487 | 11 | 1677 | 19416 | 4.2101 | 1.0000 | 0.6716 | 0.6716 |
| GO:0009166~nucleotide catabolic process | 3 | PKM, ENO1, TYMP | 0.0017 | 11 | 122 | 19416 | 43.4039 | 0.6679 | 0.5091 | 0.5091 |
| GO:1901292~nucleoside phosphate catabolic process | 3 | PKM, ENO1, TYMP | 0.0024 | 11 | 144 | 19416 | 36.7727 | 0.7833 | 0.5091 | 0.5091 |
| GO:0046434~organophosphate catabolic process | 3 | PKM, ENO1, TYMP | 0.0044 | 11 | 198 | 19416 | 26.7438 | 0.9425 | 0.6484 | 0.6484 |
| GO:1901136~carbohydrate derivative catabolic process | 3 | PKM, ENO1, TYMP | 0.0051 | 11 | 213 | 19416 | 24.8604 | 0.9629 | 0.6484 | 0.6484 |

| **Category/Term** | **Count** | **Genes** | ***p*-value** | **List total** | **Pop hits** | **Pop total** | **Fold enrichment** | **Bonferroni** | **Benjamini** | **FDR** |
| --- | --- | --- | --- | --- | --- | --- | --- | --- | --- | --- |
| **Biological process** |  |  |  |  |  |  |  |  |  |  |
| GO:0034655~nucleobase-containing compound catabolic process | 3 | PKM, ENO1, TYMP | 0.0132 | 11 | 349 | 19416 | 15.1727 | 0.9998 | 0.6484 | 0.6484 |
| GO:0009117~nucleotide metabolic process | 3 | PKM, ENO1, TYMP | 0.0219 | 11 | 456 | 19416 | 11.6124 | 1.0000 | 0.6484 | 0.6484 |
| GO:0044057~regulation of system process | 3 | ENO1, TYMP, SOD1 | 0.0358 | 11 | 595 | 19416 | 8.8996 | 1.0000 | 0.6484 | 0.6484 |
| GO:0006753~nucleoside phosphate metabolic process | 3 | PKM, ENO1, TYMP | 0.0367 | 11 | 603 | 19416 | 8.7815 | 1.0000 | 0.6484 | 0.6484 |
| GO:0055086~nucleobase-containing small molecule metabolic process | 3 | PKM, ENO1, TYMP | 0.0401 | 11 | 633 | 19416 | 8.3654 | 1.0000 | 0.6484 | 0.6484 |
| GO:0061718~glucose catabolic process to pyruvate | 2 | PKM, ENO1 | 0.0108 | 11 | 21 | 19416 | 168.1039 | 0.9991 | 0.6484 | 0.6484 |
| GO:0061621~canonical glycolysis | 2 | PKM, ENO1 | 0.0108 | 11 | 21 | 19416 | 168.1039 | 0.9991 | 0.6484 | 0.6484 |
| GO:0061615~glycolytic process through fructose-6-phosphate | 2 | PKM, ENO1 | 0.0118 | 11 | 23 | 19416 | 153.4862 | 0.9995 | 0.6484 | 0.6484 |
| GO:0061620~glycolytic process through glucose-6-phosphate | 2 | PKM, ENO1 | 0.0118 | 11 | 23 | 19416 | 153.4862 | 0.9995 | 0.6484 | 0.6484 |
| GO:0006007~glucose catabolic process | 2 | PKM, ENO1 | 0.0143 | 11 | 28 | 19416 | 126.0779 | 0.9999 | 0.6484 | 0.6484 |
| GO:0006734~NADH metabolic process | 2 | PKM, ENO1 | 0.0219 | 11 | 43 | 19416 | 82.0973 | 1.0000 | 0.6484 | 0.6484 |
| GO:0019320~hexose catabolic process | 2 | PKM, ENO1 | 0.0270 | 11 | 53 | 19416 | 66.6072 | 1.0000 | 0.6484 | 0.6484 |
| GO:0006096~glycolytic process | 2 | PKM, ENO1 | 0.0280 | 11 | 55 | 19416 | 64.1851 | 1.0000 | 0.6484 | 0.6484 |
| GO:0046365~monosaccharide catabolic process | 2 | PKM, ENO1 | 0.0290 | 11 | 57 | 19416 | 61.9330 | 1.0000 | 0.6484 | 0.6484 |
| GO:0046032~ADP catabolic process | 2 | PKM, ENO1 | 0.0290 | 11 | 57 | 19416 | 61.9330 | 1.0000 | 0.6484 | 0.6484 |
| GO:0019364~pyridine nucleotide catabolic process | 2 | PKM, ENO1 | 0.0300 | 11 | 59 | 19416 | 59.8336 | 1.0000 | 0.6484 | 0.6484 |
| GO:0009181~purine ribonucleoside diphosphate catabolic process | 2 | PKM, ENO1 | 0.0305 | 11 | 60 | 19416 | 58.8364 | 1.0000 | 0.6484 | 0.6484 |
| GO:0009137~purine nucleoside diphosphate catabolic process | 2 | PKM, ENO1 | 0.0305 | 11 | 60 | 19416 | 58.8364 | 1.0000 | 0.6484 | 0.6484 |

| **Category/Term** | **Count** | **Genes** | ***p*-value** | **List total** | **Pop hits** | **Pop total** | **Fold enrichment** | **Bonferroni** | **Benjamini** | **FDR** |
| --- | --- | --- | --- | --- | --- | --- | --- | --- | --- | --- |
| **Biological process** |  |  |  |  |  |  |  |  |  |  |
| GO:0072526~pyridine-containing compound catabolic process | 2 | PKM, ENO1 | 0.0315 | 11 | 62 | 19416 | 56.9384 | 1.0000 | 0.6484 | 0.6484 |
| GO:0009191~ribonucleoside diphosphate catabolic process | 2 | PKM, ENO1 | 0.0320 | 11 | 63 | 19416 | 56.0346 | 1.0000 | 0.6484 | 0.6484 |
| GO:0046031~ADP metabolic process | 2 | PKM, ENO1 | 0.0325 | 11 | 64 | 19416 | 55.1591 | 1.0000 | 0.6484 | 0.6484 |
| GO:0009134~nucleoside diphosphate catabolic process | 2 | PKM, ENO1 | 0.0340 | 11 | 67 | 19416 | 52.6893 | 1.0000 | 0.6484 | 0.6484 |
| GO:0009135~purine nucleoside diphosphate metabolic process | 2 | PKM, ENO1 | 0.0385 | 11 | 76 | 19416 | 46.4498 | 1.0000 | 0.6484 | 0.6484 |
| GO:0009179~purine ribonucleoside diphosphate metabolic process | 2 | PKM, ENO1 | 0.0385 | 11 | 76 | 19416 | 46.4498 | 1.0000 | 0.6484 | 0.6484 |
| GO:2000242~negative regulation of reproductive process | 2 | TIMP1, SOD1 | 0.0385 | 11 | 76 | 19416 | 46.4498 | 1.0000 | 0.6484 | 0.6484 |
| GO:0006090~pyruvate metabolic process | 2 | PKM, ENO1 | 0.0400 | 11 | 79 | 19416 | 44.6858 | 1.0000 | 0.6484 | 0.6484 |
| GO:0009185~ribonucleoside diphosphate metabolic process | 2 | PKM, ENO1 | 0.0439 | 11 | 87 | 19416 | 40.5768 | 1.0000 | 0.6673 | 0.6673 |
| GO:0009154~purine ribonucleotide catabolic process | 2 | PKM, ENO1 | 0.0444 | 11 | 88 | 19416 | 40.1157 | 1.0000 | 0.6673 | 0.6673 |
| GO:0009132~nucleoside diphosphate metabolic process | 2 | PKM, ENO1 | 0.0474 | 11 | 94 | 19416 | 37.5551 | 1.0000 | 0.6716 | 0.6716 |
| GO:0009261~ribonucleotide catabolic process | 2 | PKM, ENO1 | 0.0479 | 11 | 95 | 19416 | 37.1598 | 1.0000 | 0.6716 | 0.6716 |
| GO:0006195~purine nucleotide catabolic process | 2 | PKM, ENO1 | 0.0489 | 11 | 97 | 19416 | 36.3936 | 1.0000 | 0.6716 | 0.6716 |
| **Cellular compartment** |  |  |  |  |  |  |  |  |  |  |
| GO:0070062~extracellular exosome | 11 | LGALS3BP, PKM, IGKC, LEG1, MUC7, LCN2, DEFA3, A1BG, TIMP1, ENO1, SOD1 | 2.12E-09 | 12 | 2242 | 20747 | 8.4826 | 2.33E-07 | 9.12E-08 | 7.80E-08 |
| GO:1903561~extracellular vesicle | 11 | LGALS3BP, PKM, IGKC, LEG1, MUC7, LCN2, DEFA3, A1BG, TIMP1, ENO1, SOD1 | 3.30E-09 | 12 | 2345 | 20747 | 8.1101 | 3.63E-07 | 9.12E-08 | 7.80E-08 |

| **Category/Term** | **Count** | **Genes** | ***p*-value** | **List total** | **Pop hits** | **Pop total** | **Fold enrichment** | **Bonferroni** | **Benjamini** | **FDR** |
| --- | --- | --- | --- | --- | --- | --- | --- | --- | --- | --- |
| **Cellular compartment** |  |  |  |  |  |  |  |  |  |  |
| GO:0065010~extracellular membrane-bounded organelle | 11 | LGALS3BP, PKM, IGKC, LEG1, MUC7, LCN2, DEFA3, A1BG, TIMP1, ENO1, SOD1 | 3.32E-09 | 12 | 2346 | 20747 | 8.1066 | 3.65E-07 | 9.12E-08 | 7.80E-08 |
| GO:0043230~extracellular organelle | 11 | LGALS3BP, PKM, IGKC, LEG1, MUC7, LCN2, DEFA3, A1BG, TIMP1, ENO1, SOD1 | 3.32E-09 | 12 | 2346 | 20747 | 8.1066 | 3.65E-07 | 9.12E-08 | 7.80E-08 |
| GO:0005615~extracellular space | 11 | LGALS3BP, PKM, IGKC, LEG1, MUC7, LCN2, DEFA3, A1BG, TIMP1, ENO1, SOD1 | 2.31E-07 | 12 | 3607 | 20747 | 5.2725 | 2.54E-05 | 5.09E-06 | 4.35E-06 |
| GO:0031982~vesicle | 11 | LGALS3BP, PKM, IGKC, LEG1, MUC7, LCN2, DEFA3, A1BG, TIMP1, ENO1, SOD1 | 1.28E-06 | 12 | 4296 | 20747 | 4.4269 | 1.41E-04 | 1.57E-05 | 1.34E-05 |
| GO:0005576~extracellular region | 11 | LGALS3BP, PKM, IGKC, LEG1, MUC7, LCN2, DEFA3, A1BG, TIMP1, ENO1, SOD1 | 3.37E-06 | 12 | 4743 | 20747 | 4.0097 | 3.71E-04 | 3.71E-05 | 3.17E-05 |
| GO:0012505~endomembrane system | 8 | LGALS3BP, PKM, MUC7, LCN2, DEFA3, A1BG, TIMP1, ENO1 | 0.0070 | 12 | 5117 | 20747 | 2.7030 | 0.5365 | 0.0403 | 0.0345 |
| GO:0043233~organelle lumen | 8 | LGALS3BP, PKM, MUC7, LCN2, DEFA3, A1BG, TIMP1, SOD1 | 0.0261 | 12 | 6439 | 20747 | 2.1481 | 0.9454 | 0.1305 | 0.1115 |
| GO:0070013~intracellular organelle lumen | 8 | LGALS3BP, PKM, MUC7, LCN2, DEFA3, A1BG, TIMP1, SOD1 | 0.0261 | 12 | 6439 | 20747 | 2.1481 | 0.9454 | 0.1305 | 0.1115 |
| GO:0031974~membrane-enclosed lumen | 8 | LGALS3BP, PKM, MUC7, LCN2, DEFA3, A1BG, TIMP1, SOD1 | 0.0261 | 12 | 6439 | 20747 | 2.1481 | 0.9454 | 0.1305 | 0.1115 |
| GO:0071944~cell periphery | 8 | LGALS3BP, PKM, IGKC, MUC7, A1BG, TIMP1, ENO1, SOD1 | 0.0335 | 12 | 6739 | 20747 | 2.0524 | 0.9765 | 0.1604 | 0.1370 |
| GO:0031410~cytoplasmic vesicle | 7 | LGALS3BP, PKM, LCN2, DEFA3, A1BG, TIMP1, SOD1 | 0.0011 | 12 | 2625 | 20747 | 4.6104 | 0.1108 | 0.0085 | 0.0073 |
| GO:0097708~intracellular vesicle | 7 | LGALS3BP, PKM, LCN2, DEFA3, A1BG, TIMP1, SOD1 | 0.0011 | 12 | 2634 | 20747 | 4.5947 | 0.1127 | 0.0085 | 0.0073 |
| GO:0034774~secretory granule lumen | 6 | LGALS3BP, PKM, LCN2, DEFA3, A1BG, TIMP1 | 4.16E-07 | 12 | 329 | 20747 | 31.5304 | 0.0000457 | 0.0000062 | 0.0000053 |

| **Category/Term** | **Count** | **Genes** | ***p*-value** | **List total** | **Pop hits** | **Pop total** | **Fold enrichment** | **Bonferroni** | **Benjamini** | **FDR** |
| --- | --- | --- | --- | --- | --- | --- | --- | --- | --- | --- |
| **Cellular compartment** |  |  |  |  |  |  |  |  |  |  |
| GO:0060205~cytoplasmic vesicle lumen | 6 | LGALS3BP, PKM, LCN2, DEFA3, A1BG, TIMP1 | 4.48E-07 | 12 | 334 | 20747 | 31.0584 | 0.0000493 | 0.0000062 | 0.0000053 |
| GO:0031983~vesicle lumen | 6 | LGALS3BP, PKM, LCN2, DEFA3, A1BG, TIMP1 | 4.54E-07 | 12 | 335 | 20747 | 30.9657 | 0.0000500 | 0.0000062 | 0.0000053 |
| GO:0030141~secretory granule | 6 | LGALS3BP, PKM, LCN2, DEFA3, A1BG, TIMP1 | 6.67E-05 | 12 | 932 | 20747 | 11.1304 | 0.0073 | 0.0007 | 0.0006 |
| GO:0099503~secretory vesicle | 6 | LGALS3BP, PKM, LCN2, DEFA3, A1BG, TIMP1 | 1.62E-04 | 12 | 1124 | 20747 | 9.2291 | 0.0177 | 0.0015 | 0.0013 |
| GO:0062023~collagen-containing extracellular matrix | 4 | LGALS3BP, PKM, A1BG, TIMP1 | 0.0014 | 12 | 438 | 20747 | 15.7892 | 0.1389 | 0.0100 | 0.0085 |
| GO:0031012~extracellular matrix | 4 | LGALS3BP, PKM, A1BG, TIMP1 | 0.0030 | 12 | 576 | 20747 | 12.0064 | 0.2793 | 0.0183 | 0.0156 |
| GO:0030312~external encapsulating structure | 4 | LGALS3BP, PKM, A1BG, TIMP1 | 0.0030 | 12 | 577 | 20747 | 11.9856 | 0.2805 | 0.0183 | 0.0156 |
| GO:0072562~blood microparticle | 3 | LGALS3BP, IGKC, A1BG | 0.0028 | 12 | 151 | 20747 | 34.3493 | 0.2631 | 0.0183 | 0.0156 |
| GO:0031093~platelet alpha granule lumen | 2 | A1BG, TIMP1 | 0.0355 | 12 | 68 | 20747 | 50.8505 | 0.9812 | 0.1626 | 0.1390 |
| GO:0031091~platelet alpha granule | 2 | A1BG, TIMP1 | 0.0477 | 12 | 92 | 20747 | 37.5851 | 0.9954 | 0.2100 | 0.1794 |
| **Molecular function** |  |  |  |  |  |  |  |  |  |  |
| GO:0042802~identical protein binding | 6 | PKM, LCN2, DEFA3, ENO1, TYMP, SOD1 | 0.0034 | 11 | 2269 | 19202 | 4.6161 | 0.3379 | 0.1372 | 0.1372 |
| GO:0042803~protein homodimerization activity | 5 | PKM, DEFA3, ENO1, TYMP, SOD1 | 4.08E-04 | 11 | 753 | 19202 | 11.5912 | 0.0474 | 0.0485 | 0.0485 |
| GO:0046983~protein dimerization activity | 5 | PKM, DEFA3, ENO1, TYMP, SOD1 | 0.0021 | 11 | 1165 | 19202 | 7.4920 | 0.2219 | 0.1253 | 0.1253 |

**Supplementary Table 3.** **Pathway profiles of 12 upregulated genes in the MF and CF groups.**

| **Category/Term** | **Count** | **Genes** | ***p*-value** | **List total** | **Pop hits** | **Pop total** | **Fold enrichment** | **Bonferroni** | **Benjamini** | **FDR** |
| --- | --- | --- | --- | --- | --- | --- | --- | --- | --- | --- |
| **Reactome pathway** |  |  |  |  |  |  |  |  |  |  |
| R-HSA-168256~Immune System | 8 | PKM, IGKC, MUC7, LCN2, DEFA3, A1BG, TIMP1, SOD1 | 6.07E-04 | 11 | 2177 | 11494 | 3.8398 | 0.0583 | 0.0200 | 0.0200 |
| R-HSA-168249~Innate Immune System | 6 | PKM, IGKC, MUC7, LCN2, DEFA3, A1BG | 0.0016 | 11 | 1150 | 11494 | 5.4517 | 0.1490 | 0.0323 | 0.0323 |
| R-HSA-109582~Hemostasis | 5 | LGALS3BP, IGKC, A1BG, TIMP1, SOD1 | 0.0021 | 11 | 699 | 11494 | 7.4743 | 0.1894 | 0.0350 | 0.0350 |
| R-HSA-114608~Platelet degranulation | 4 | LGALS3BP, A1BG, TIMP1, SOD1 | 1.64E-04 | 11 | 131 | 11494 | 31.9056 | 0.0161 | 0.0091 | 0.0091 |
| R-HSA-76005~Response to elevated platelet cytosolic Ca2+ | 4 | LGALS3BP, A1BG, TIMP1, SOD1 | 1.83E-04 | 11 | 136 | 11494 | 30.7326 | 0.0180 | 0.0091 | 0.0091 |
| R-HSA-76002~Platelet activation, signaling and aggregation | 4 | LGALS3BP, A1BG, TIMP1, SOD1 | 0.0013 | 11 | 266 | 11494 | 15.7129 | 0.1212 | 0.0323 | 0.0323 |
| **Wiki pathway** |  |  |  |  |  |  |  |  |  |  |
| WP5049:Glycolysis in senescence | 2 | PKM, ENO1 | 0.0074 | 7 | 11 | 8832 | 229.4026 | 0.2911 | 0.1350 | 0.1350 |
| WP4629:Aerobic glycolysis | 2 | PKM, ENO1 | 0.0081 | 7 | 12 | 8832 | 210.2857 | 0.3130 | 0.1350 | 0.1350 |
| WP4628:Aerobic glycolysis augmented | 2 | PKM, ENO1 | 0.0088 | 7 | 13 | 8832 | 194.1099 | 0.3341 | 0.1350 | 0.1350 |
| WP4290:Metabolic reprogramming in colon cancer | 2 | PKM, ENO1 | 0.0282 | 7 | 42 | 8832 | 60.0816 | 0.7318 | 0.2778 | 0.2778 |
| WP534:Glycolysis and gluconeogenesis | 2 | PKM, ENO1 | 0.0301 | 7 | 45 | 8832 | 56.0762 | 0.7559 | 0.2778 | 0.2778 |
| WP5348:11p11 2 copy number variation syndrome | 2 | LGALS3BP, TIMP1 | 0.0374 | 7 | 56 | 8832 | 45.0612 | 0.8273 | 0.2872 | 0.2872 |

**Supplementary Table 4.** **Mean log_2_ fold-change values of 37 proteins exclusively identified in the MF group.**

| **Protein name** | **Gene** | **Mean Value** | **UniProtKB Entry** |
| --- | --- | --- | --- |
| Immunoglobulin heavy constant alpha 2 | IGHA2 | -0.07275 | P01877 |
| Immunoglobulin heavy constant gamma 3 | IGHG3 | 0.02680 | P01860 |
| Immunoglobulin heavy constant gamma 2 | IGHG2 | 0.24057 | P01859 |
| Isoform 3 of Cysteine-rich secretory protein 3 | CRISP3 | -0.15902 | P54108-3 |
| Cytidine deaminase | CDA | -0.56677 | P32320 |
| Heat shock 70 kDa protein 6 | HSPA6 | -0.91720 | P17066 |
| Immunoglobulin heavy variable 3-74 | IGHV3-74 | 0.83976 | A0A0B4J1X5 |
| Complement C3 | C3 | -0.34625 | P01024 |
| Rab GDP dissociation inhibitor beta | GDI2 | 0.14037 | P50395 |
| 14-3-3 protein sigma | SFN | 0.92530 | P31947 |
| Immunoglobulin heavy variable 4-39 | IGHV4-39 | 0.11004 | P01824 |
| Profilin-1 | PFN1 | 0.20769 | P07737 |
| Low affinity immunoglobulin gamma Fc region receptor III-A | FCGR3A | 0.80622 | P08637 |
| Immunoglobulin lambda variable 2-18 | IGLV2-18 | -0.17473 | A0A075B6J9 |
| Immunoglobulin lambda variable 7-46 | IGLV7-46 | 0.52269 | A0A075B6I9 |
| Apolipoprotein A-I | APOA1 | 0.04612 | P02647 |
| Immunoglobulin kappa variable 4-1 | IGKV4-1 | 0.21526 | P06312 |
| SPARC-like protein 1 | SPARCL1 | -1.04889 | Q14515 |
| Isoform 3 of Actin-related protein 2/3 complex subunit 4 | ARPC4 | 0.37907 | P59998-3 |
| Isoform 2 of Neutral alpha-glucosidase AB | GANAB | -0.36014 | Q14697-2 |
| 14-3-3 protein zeta/delta | YWHAZ | 0.42309 | P63104 |
| Isoform 2 of Adenylosuccinate synthetase isozyme 1 (Adenylosuccinate synthetase isozyme 1) | ADSSL1 (ADSS1) | 6.80083 | Q8N142-2 |
| Immunoglobulin lambda variable 2-14 | IGLV2-14 | 0.53039 | P01704 |
| Heat shock cognate 71 kDa protein | HSPA8 | -0.02502 | P11142 |
| Immunoglobulin heavy variable 6-1 | IGHV6-1 | -0.12856 | A0A0B4J1U7 |
| Puromycin-sensitive aminopeptidase | NPEPPS | -0.37749 | P55786 |
| Desmoglein-1 | DSG1 | -0.02481 | Q02413 |
| Immunoglobulin lambda variable 1-44 | IGLV1-44 | 0.22278 | P01699 |
| Immunoglobulin kappa variable 1D-33 | IGKV1D-33 | -0.65575 | P01593 |
| Ezrin | EZR | -1.32111 | P15311 |
| Alpha-1-antitrypsin | SERPINA1 | 0.32651 | P01009 |
| Immunoglobulin kappa variable 2D-29 | IGKV2D-29 | 1.00941 | A0A075B6S2 |
| Myeloblastin | PRTN3 | -0.25103 | P24158 |
| Serpin B3 | SERPINB3 | 0.64136 | P29508 |
| Serpin B5 | SERPINB5 | 0.87147 | P36952 |
| Immunoglobulin lambda-like polypeptide 1 | IGLL1 | 1.23061 | P15814 |
| Fibrinogen gamma chain | FGG | -0.21660 | P02679 |

**Supplementary Table 5.** **Key biological function of 37 proteins exclusively identified in the MF group.**

| **UniProtKB Entry** | **Gene** | **Protein name** | **Key biological function** |
| --- | --- | --- | --- |
| P01877 | IGHA2 | Immunoglobulin heavy constant alpha 2 | Negative regulation of complement activation, lectin pathway, adaptive immune response, acute inflammatory response to antigenic stimulus, acute-phase response, immune response, complement activation, classical pathway, response to nutrient, response to carbon dioxide, antibacterial humoral response, response to prostaglandin E, stem cell differentiation, B cell receptor signaling pathway, response to glucocorticoid, positive regulation of respiratory burst |
| P01860 | IGHG3 | Immunoglobulin heavy constant gamma 3 | Adaptive immune response, complement activation, classical pathway, antibacterial humoral response, B cell receptor signaling pathway |
| P01859 | IGHG2 | Immunoglobulin heavy constant gamma 2 | Adaptive immune response, complement activation, classical pathway, antibacterial humoral response, B cell receptor signaling pathway |
| P54108-3 | CRISP3 | Isoform 3 of Cysteine-rich secretory protein 3 | Defense response, sexual reproduction, innate immune response, |
| P32320 | CDA | Cytidine deaminase | Cell surface receptor signaling pathway, pyrimidine-containing compound salvage, cytosine metabolic process, UMP salvage, negative regulation of nucleotide metabolic process, response to cycloheximide, cellular response to external biotic stimulus, pyrimidine-containing compound metabolic process, carbohydrate derivative metabolic process, |
| P17066 | HSPA6 | Heat shock 70 kDa protein 6 | Response to unfolded protein, cellular response to heat, protein refolding, chaperone cofactor-dependent protein refolding |
| A0A0B4J1X5 | IGHV3-74 | Immunoglobulin heavy variable 3-74 | Immunoglobulin mediated immune response |
| P01024 | C3 | Complement C3 | Positive regulation of protein phosphorylation, fatty acid metabolic process, inflammatory response, complement activation, signal transduction, G protein-coupled receptor signaling pathway, response to bacterium, positive regulation of vascular endothelial growth factor production, positive regulation of D-glucose transmembrane transport, regulation of triglyceride biosynthetic process, positive regulation of lipid storage, B cell activation, positive regulation of G protein-coupled receptor signaling pathway, positive regulation of angiogenesis, positive regulation of receptor-mediated endocytosis, positive regulation of phagocytosis, engulfment, amyloid-beta clearance, complement-dependent cytotoxicity, complement-mediated synapse pruning, positive regulation of apoptotic cell clearance |
| P50395 | GDI2 | Rab GDP dissociation inhibitor beta | Signal transduction, small GTPase-mediated signal transduction, protein transport, vesicle-mediated transport, negative regulation of cilium assembly, negative regulation of protein localization to cilium |
| P31947 | SFN | 14-3-3 protein sigma | Negative regulation of transcription by RNA polymerase II, release of cytochrome c from mitochondria, keratinocyte development, negative regulation of protein kinase activity, signal transduction, protein localization, intrinsic apoptotic signaling pathway in response to DNA damage, regulation of epidermal cell division, regulation of cell-cell adhesion, positive regulation of cell growth, keratinization, regulation of protein localization, positive regulation of epidermal cell differentiation, negative regulation of innate immune response,regulation of cell cycle, establishment of skin barrier, stem cell proliferation, cAMP/PKA signal transduction, negative regulation of protein localization to plasma membrane, positive regulation of protein localization, negative regulation of stem cell proliferation |

| **UniProtKB Entry** | **Gene** | **Protein name** | **Key biological function** |
| --- | --- | --- | --- |
| P01824 | IGHV4-39 | Immunoglobulin heavy variable 4-39 | Adaptive immune response, immune response, immunoglobulin mediated immune response |
| P07737 | PFN1 | Profilin-1 | Neural tube closure, regulation of transcription by RNA polymerase II, positive regulation of epithelial cell migration, actin cytoskeleton organization, regulation of actin filament polymerization, positive regulation of ATP-dependent activity, modulation of chemical synaptic transmission, protein stabilization |
| P08637 | FCGR3A | Low affinity immunoglobulin gamma Fc region receptor III-A | Antibody-dependent cellular cytotoxicity, immune response, cell surface receptor signaling pathway, calcium-mediated signaling, natural killer cell activation, positive regulation of tumor necrosis factor production, Fc-gamma receptor signaling pathway, macrophage activation, natural killer cell mediated cytotoxicity, natural killer cell degranulation, phosphatidylinositol 3-kinase/protein kinase B signal transduction |
| A0A075B6J9 | IGLV2-18 | Immunoglobulin lambda variable 2-18 | Adaptive immune response, immune response |
| A0A075B6I9 | IGLV7-46 | Immunoglobulin lambda variable 7-46 | Adaptive immune response, immune response |
| P02647 | APOA1 | Apolipoprotein A-I | Endothelial cell proliferation, negative regulation of cytokine production involved in immune response, phospholipid and cholesterol metabolic process, phosphatidylcholine biosynthetic process, lipid transport, G protein-coupled receptor signaling pathway, integrin-mediated signaling pathway, glucocorticoid metabolic process, protein oxidation, peptidyl-methionine modification, lipid storage, regulation of Cdc42 protein signal transduction, negative regulation of interleukin-1 beta production, cholesterol and phospholipid efflux, negative regulation of cell-cell adhesion, amyloid-beta formation, high-density lipoprotein particle remodeling, high-density lipoprotein particle assembly, high-density lipoprotein particle clearance, positive regulation of Rho protein signal transduction, lipoprotein metabolic process, cholesterol homeostasis, blood vessel endothelial cell migration, positive regulation of phagocytosis, protein stabilization, negative chemotaxis, vitamin transport, positive regulation of hydrolase activity, phospholipid homeostasis, negative regulation of cell adhesion molecule production, triglyceride homeostasis, cholesterol import, cellular response to lipoprotein particle stimulus, positive regulation of cholesterol metabolic process, positive regulation of substrate adhesion-dependent cell spreading, |
| P06312 | IGKV4-1 | Immunoglobulin kappa variable 4-1 | Adaptive immune response, immune response |
| Q14515 | SPARCL1 | SPARC-like protein 1 | Signal transduction, regulation of synapse organization, synaptic membrane adhesion |
| P59998-3 | ARPC4 | Isoform 3 of Actin-related protein 2/3 complex subunit 4 | Actin filament polymerization, Arp2/3 complex-mediated actin nucleation, protein modification process, actin nucleation |
| Q14697-2 | GANAB | Isoform 2 of Neutral alpha-glucosidase AB | Carbohydrate metabolic process, N-glycan processing, |

| **UniProtKB Entry** | **Gene** | **Protein name** | **Key biological function** |
| --- | --- | --- | --- |
| P63104 | YWHAZ | 14-3-3 protein zeta/delta | Angiogenesis, protein phosphorylation, protein targeting, signal transduction, synaptic target recognition, protein localization, regulation of protein stability, cellular response to glucose starvation, negative regulation of apoptotic process, regulation of programmed cell death, negative regulation of innate immune response, establishment of Golgi localization, ERK_1_ and ERK_2_ cascade, regulation of ERK1 and ERK2 cascade, regulation of synapse maturation, Golgi reassembly, negative regulation of protein localization to nucleus, negative regulation of TORC1 signaling, |
| Q8N142-2 | ADSSL1 | Isoform 2 of Adenylosuccinate synthetase isozyme 1 | Immune system process, purine nucleotide biosynthetic process, AMP biosynthetic process, aspartate metabolic process, glutamine metabolic process, 'de novo' AMP biosynthetic process, IMP metabolic process, cellular response to electrical stimulus, cellular response to xenobiotic stimulus |
| P01704 | IGLV2-14 | Immunoglobulin lambda variable 2-14 | Adaptive immune response, immune response |
| P11142 | HSPA8 | Heat shock cognate 71 kDa protein | mRNA splicing, via spliceosome, protein folding, response to unfolded protein, signal transduction, RNA splicing, cellular response to starvation, positive regulation of cell migration, regulation of protein stability, protein refolding, regulation of protein-containing complex assembly, negative regulation of DNA-templated transcription, ATP metabolic process, chaperone cofactor-dependent protein refolding, membrane organization, regulation of protein complex stability, chaperone-mediated autophagy, protein targeting to lysosome involved in chaperone-mediated autophagy, cellular response to steroid hormone stimulus, clathrin coat disassembly, negative regulation of NLRP3 inflammasome complex assembly, negative regulation of supramolecular fiber organization, regulation of protein import, chaperone-mediated autophagy translocation complex disassembly |
| A0A0B4J1U7 | IGHV6-1 | Immunoglobulin heavy variable 6-1 | Immunoglobulin mediated immune response |
| P55786 | NPEPPS | Puromycin-sensitive aminopeptidase | Protein polyubiquitination, proteolysis, peptide catabolic process, cellular response to hypoxia, positive regulation of protein targeting to mitochondrion |
| Q02413 | DSG1 | Desmoglein-1 | Cell-cell junction assembly, homophilic cell adhesion, calcium-dependent cell-cell adhesion via plasma membrane cell adhesion molecules, response to progesterone, protein stabilization, cell-cell adhesion |
| P01699 | IGLV1-44 | Immunoglobulin lambda variable 1-44 | Adaptive immune response, immune response |
| P01593 | IGKV1D-33 | Immunoglobulin kappa variable 1D-33 | Adaptive immune response, immune response |
| P01009 | SERPINA1 | Alpha-1-antitrypsin | Acute-phase response, blood coagulation, |

| **UniProtKB Entry** | **Gene** | **Protein name** | **Key biological function** |
| --- | --- | --- | --- |
| P15311 | EZR | Ezrin | Sphingosine-1-phosphate receptor signaling pathway, leukocyte cell-cell adhesion, regulation of cell shape, positive regulation of gene expression, protein kinase A signaling, membrane to membrane docking, microvillus assembly, actin cytoskeleton organization, astral microtubule organization, protein-containing complex localization, receptor internalization, negative regulation of interleukin-2 production, establishment or maintenance of apical/basal cell polarity, positive regulation of multicellular organism growth, cortical microtubule organization, positive regulation of protein catabolic process, filopodium assembly, negative regulation of T cell receptor signaling pathway, actin filament bundle assembly, regulation of cellular component organization, establishment of centrosome localization, establishment of endothelial barrier, negative regulation of ERK1 and ERK2 cascade, cellular response to cAMP, protein localization to plasma membrane, regulation of organelle assembly, positive regulation of protein localization to early endosome, positive regulation of protein localization to plasma membrane, negative regulation of p38MAPK cascade, positive regulation of early endosome to late endosome transport |
| A0A075B6S2 | IGKV2D-29 | Immunoglobulin kappa variable 2D-29 | Adaptive immune response, immune response |
| P24158 | PRTN3 | Myeloblastin | Proteolysis, membrane protein ectodomain proteolysis, positive regulation of cell population proliferation, antimicrobial humoral response, collagen catabolic process, positive regulation of GTPase activity, cell-cell junction maintenance, negative regulation of phagocytosis, neutrophil extravasation, mature conventional dendritic cell differentiation |
| P29508 | SERPINB3 | Serpin B3 | Positive regulation of cell population proliferation, negative regulation of peptidase activity, positive regulation of epithelial to mesenchymal transition, positive regulation of endopeptidase activity, negative regulation of endopeptidase activity, positive regulation of cell migration, autocrine signaling, paracrine signaling, negative regulation of catalytic activity, negative regulation of JUN kinase activity, negative regulation of proteolysis, symbiont entry into host cell |
| P36952 | SERPINB5 | Serpin B5 | Morphogenesis of an epithelium, extracellular matrix organization, regulation of epithelial cell proliferation, prostate gland morphogenesis |
| P15814 | IGLL1 | Immunoglobulin lambda-like polypeptide 1 | Immune response, immunoglobulin mediated immune response |
| P02679 | FGG | Fibrinogen gamma chain | Cell-matrix adhesion, protein secretion, platelet activation, plasminogen activation, positive regulation of heterotypic cell-cell adhesion, platelet maturation, fibrinolysis, positive regulation of vasoconstriction, positive regulation of exocytosis, positive regulation of protein secretion, protein polymerization, response to calcium ion, protein-containing complex assembly, positive regulation of ERK1 and ERK2 cascade, platelet aggregation, cellular response to interleukin-1, cellular response to interleukin-6, blood coagulation, fibrin clot formation, positive regulation of peptide hormone secretion, positive regulation of substrate adhesion-dependent cell spreading, negative regulation of extrinsic apoptotic signaling pathway via death domain receptors, negative regulation of endothelial cell apoptotic process |

**Supplementary Table 6.** **Gene Ontology profiles of 37 genes exclusively identified in the MF group.**

| **Category/Term** | **Count** | **Genes** | ***p*-value** | **List total** | **Pop hits** | **Pop total** | **Fold enrichment** | **Bonferroni** | **Benjamini** | **FDR** |
| --- | --- | --- | --- | --- | --- | --- | --- | --- | --- | --- |
| **Biological process** |  |  |  |  |  |  |  |  |  |  |
| GO:0050896~response to stimulus | 32 | CDA, SERPINA1, IGHV6-1, CRISP3, GDI2, IGHV4-39, C3, IGHG3, NPEPPS, FCGR3A, IGHG2, IGLV2-14, IGLV7-46, IGLV1-44, IGLV2-18, SFN, IGHA2, HSPA8, IGHV3-74, FGG, HSPA6, APOA1, IGKV1D-33, YWHAZ, IGKV2D-29, IGLL1, IGKV4-1, SPARCL1, DSG1, PRTN3, ADSS1, EZR | 2.31E-07 | 37 | 8582 | 19416 | 1.9567 | 2.78E-04 | 3.09E-05 | 3.06E-05 |
| GO:0002376~immune system process | 19 | IGHV6-1, IGHV3-74, CRISP3, IGHV4-39, IGKV1D-33, C3, IGHG3, FCGR3A, IGKV2D-29, IGHG2, IGLV2-14, IGLL1, IGLV7-46, IGLV1-44, IGKV4-1, IGLV2-18, PRTN3, ADSS1, IGHA2 | 8.58E-08 | 37 | 2511 | 19416 | 3.9707 | 1.03E-04 | 1.47E-05 | 1.46E-05 |
| GO:0006955~immune response | 18 | IGHV6-1, IGHV3-74, CRISP3, IGHV4-39, IGKV1D-33, C3, IGHG3, FCGR3A, IGKV2D-29, IGHG2, IGLV2-14, IGLL1, IGLV7-46, IGLV1-44, IGKV4-1, IGLV2-18, PRTN3, IGHA2 | 5.49E-09 | 37 | 1839 | 19416 | 5.1363 | 6.61E-06 | 1.65E-06 | 1.64E-06 |
| GO:0002250~adaptive immune response | 16 | IGHV6-1, IGHV3-74, IGHV4-39, IGKV1D-33, C3, IGHG3, FCGR3A, IGKV2D-29, IGHG2, IGLV2-14, IGLL1, IGLV7-46, IGLV1-44, IGKV4-1, IGLV2-18, IGHA2 | 7.90E-13 | 37 | 719 | 19416 | 11.6775 | 9.50E-10 | 9.50E-10 | 9.41E-10 |
| GO:0006950~response to stress | 15 | HSPA8, SERPINA1, CRISP3, HSPA6, FGG, YWHAZ, C3, IGHG3, NPEPPS, FCGR3A, IGHG2, PRTN3, SFN, ADSS1, IGHA2 | 0.0043 | 37 | 3689 | 19416 | 2.1337 | 0.9947 | 0.2612 | 0.2588 |
| GO:0051128~regulation of cellular component organization | 11 | C3, HSPA8, CDA, GDI2, SPARCL1, APOA1, PRTN3, SFN, PFN1, EZR, YWHAZ | 0.0158 | 37 | 2569 | 19416 | 2.2469 | 1 | 0.5573 | 0.5522 |
| GO:0002682~regulation of immune system process | 10 | C3, IGHG3, HSPA8, FCGR3A, IGHG2, APOA1, SFN, EZR, IGHA2, YWHAZ | 0.0039 | 37 | 1752 | 19416 | 2.9952 | 0.9906 | 0.2612 | 0.2588 |
| GO:0032879~regulation of localization | 10 | C3, HSPA8, NPEPPS, GDI2, FGG, APOA1, PRTN3, SFN, EZR, YWHAZ | 0.0143 | 37 | 2146 | 19416 | 2.4453 | 1 | 0.5377 | 0.5328 |
| GO:0016064~immunoglobulin mediated immune response | 9 | C3, IGHG3, FCGR3A, IGHV6-1, IGHV3-74, IGHG2, IGLL1, IGHV4-39, IGHA2 | 2.05E-09 | 37 | 194 | 19416 | 24.3444 | 2.46E-06 | 1.00E-06 | 9.94E-07 |
| GO:0019724~B cell mediated immunity | 9 | C3, IGHG3, FCGR3A, IGHV6-1, IGHV3-74, IGHG2, IGLL1, IGHV4-39, IGHA2 | 2.50E-09 | 37 | 199 | 19416 | 23.7327 | 3.01E-06 | 1.00E-06 | 9.94E-07 |

| **Category/Term** | **Count** | **Genes** | ***p*-value** | **List total** | **Pop hits** | **Pop total** | **Fold enrichment** | **Bonferroni** | **Benjamini** | **FDR** |
| --- | --- | --- | --- | --- | --- | --- | --- | --- | --- | --- |
| **Biological process** |  |  |  |  |  |  |  |  |  |  |
| GO:0002449~lymphocyte mediated immunity | 9 | C3, IGHG3, FCGR3A, IGHV6-1, IGHV3-74, IGHG2, IGLL1, IGHV4-39, IGHA2 | 3.23E-08 | 37 | 276 | 19416 | 17.1116 | 3.88E-05 | 7.04E-06 | 6.98E-06 |
| GO:0002460~adaptive immune response based on somatic recombination of immune receptors built from immunoglobulin superfamily domains | 9 | C3, IGHG3, FCGR3A, IGHV6-1, IGHV3-74, IGHG2, IGLL1, IGHV4-39, IGHA2 | 3.51E-08 | 37 | 279 | 19416 | 16.9276 | 4.22E-05 | 7.04E-06 | 6.98E-06 |
| GO:0002443~leukocyte mediated immunity | 9 | C3, IGHG3, FCGR3A, IGHV6-1, IGHV3-74, IGHG2, IGLL1, IGHV4-39, IGHA2 | 1.37E-07 | 37 | 333 | 19416 | 14.1826 | 1.65E-04 | 2.06E-05 | 2.04E-05 |
| GO:0002252~immune effector process | 9 | C3, IGHG3, FCGR3A, IGHV6-1, IGHV3-74, IGHG2, IGLL1, IGHV4-39, IGHA2 | 3.50E-06 | 37 | 512 | 19416 | 9.2242 | 0.0042 | 4.21E-04 | 4.17E-04 |
| GO:0050776~regulation of immune response | 9 | C3, IGHG3, HSPA8, FCGR3A, IGHG2, SFN, EZR, IGHA2, YWHAZ | 6.69E-04 | 37 | 1080 | 19416 | 4.3730 | 0.5531 | 0.0575 | 0.0570 |
| GO:0009605~response to external stimulus | 9 | C3, IGHG3, CDA, FCGR3A, IGHG2, CRISP3, APOA1, PRTN3, IGHA2 | 0.0330 | 37 | 2072 | 19416 | 2.2793 | 1.0000 | 0.8457 | 0.8380 |
| GO:0043207~response to external biotic stimulus | 8 | C3, IGHG3, CDA, FCGR3A, IGHG2, CRISP3, PRTN3, IGHA2 | 0.0211 | 37 | 1538 | 19416 | 2.7296 | 1.0000 | 0.6499 | 0.6439 |
| GO:0009607~response to biotic stimulus | 8 | C3, IGHG3, CDA, FCGR3A, IGHG2, CRISP3, PRTN3, IGHA2 | 0.0241 | 37 | 1580 | 19416 | 2.6570 | 1.0000 | 0.7048 | 0.6983 |
| GO:0006952~defense response | 8 | C3, IGHG3, FCGR3A, SERPINA1, IGHG2, CRISP3, PRTN3, IGHA2 | 0.0247 | 37 | 1589 | 19416 | 2.6419 | 1.0000 | 0.7048 | 0.6983 |
| GO:0044419~biological process involved in interspecies interaction between organisms | 8 | SERPINB3, C3, IGHG3, FCGR3A, IGHG2, CRISP3, PRTN3, IGHA2 | 0.0349 | 37 | 1707 | 19416 | 2.4593 | 1.0000 | 0.8736 | 0.8656 |
| GO:0032880~regulation of protein localization | 7 | HSPA8, NPEPPS, GDI2, FGG, SFN, EZR, YWHAZ | 0.0075 | 37 | 952 | 19416 | 3.8585 | 0.9999 | 0.3753 | 0.3718 |
| GO:0140546~defense response to symbiont | 7 | C3, IGHG3, FCGR3A, IGHG2, CRISP3, PRTN3, IGHA2 | 0.0077 | 37 | 955 | 19416 | 3.8464 | 0.9999 | 0.3753 | 0.3718 |
| GO:0060341~regulation of cellular localization | 7 | HSPA8, NPEPPS, GDI2, FGG, SFN, EZR, YWHAZ | 0.0128 | 37 | 1067 | 19416 | 3.4426 | 1.0000 | 0.4982 | 0.4937 |
| GO:0098542~defense response to other organism | 7 | C3, IGHG3, FCGR3A, IGHG2, CRISP3, PRTN3, IGHA2 | 0.0171 | 37 | 1137 | 19416 | 3.2307 | 1.0000 | 0.5806 | 0.5753 |
| GO:0032101~regulation of response to external stimulus | 7 | C3, HSPA8, FGG, APOA1, SFN, IGHA2, YWHAZ | 0.0216 | 37 | 1198 | 19416 | 3.0662 | 1.0000 | 0.6499 | 0.6439 |
| GO:0034330~cell junction organization | 6 | C3, SPARCL1, DSG1, PRTN3, PFN1, EZR | 0.0039 | 37 | 575 | 19416 | 5.4757 | 0.9914 | 0.2612 | 0.2588 |

| **Category/Term** | **Count** | **Genes** | ***p*-value** | **List total** | **Pop hits** | **Pop total** | **Fold enrichment** | **Bonferroni** | **Benjamini** | **FDR** |
| --- | --- | --- | --- | --- | --- | --- | --- | --- | --- | --- |
| **Biological process** |  |  |  |  |  |  |  |  |  |  |
| GO:0002683~negative regulation of immune system process | 6 | HSPA8, APOA1, SFN, EZR, IGHA2, YWHAZ | 0.0041 | 37 | 581 | 19416 | 5.4192 | 0.9931 | 0.2612 | 0.2588 |
| GO:0031347~regulation of defense response | 6 | C3, HSPA8, APOA1, SFN, IGHA2, YWHAZ | 0.0259 | 37 | 916 | 19416 | 3.4373 | 1.0000 | 0.7082 | 0.7018 |
| GO:0002768~immune response-regulating cell surface receptor signaling pathway | 5 | C3, IGHG3, FCGR3A, IGHG2, IGHA2 | 0.0021 | 37 | 295 | 19416 | 8.8942 | 0.9200 | 0.1682 | 0.1667 |
| GO:0006959~humoral immune response | 5 | C3, IGHG3, IGHG2, PRTN3, IGHA2 | 0.0023 | 37 | 303 | 19416 | 8.6594 | 0.9382 | 0.1738 | 0.1722 |
| GO:0031647~regulation of protein stability | 5 | HSPA8, DSG1, APOA1, PFN1, YWHAZ | 0.0079 | 37 | 428 | 19416 | 6.1303 | 0.9999 | 0.3753 | 0.3718 |
| GO:0002764~immune response-regulating signaling pathway | 5 | C3, IGHG3, FCGR3A, IGHG2, IGHA2 | 0.0081 | 37 | 432 | 19416 | 6.0736 | 0.9999 | 0.3753 | 0.3718 |
| GO:0032102~negative regulation of response to external stimulus | 5 | FGG, APOA1, SFN, IGHA2, YWHAZ | 0.0099 | 37 | 458 | 19416 | 5.7288 | 1.0000 | 0.4259 | 0.4220 |
| GO:0060627~regulation of vesicle-mediated transport | 5 | C3, FGG, APOA1, PRTN3, EZR | 0.0201 | 37 | 566 | 19416 | 4.6357 | 1.0000 | 0.6499 | 0.6439 |
| GO:0051129~negative regulation of cellular component organization | 5 | HSPA8, GDI2, APOA1, PRTN3, PFN1 | 0.0464 | 37 | 737 | 19416 | 3.5601 | 1.0000 | 1.0000 | 0.9917 |
| GO:0006958~complement activation, classical pathway | 4 | C3, IGHG3, IGHG2, IGHA2 | 8.97E-05 | 37 | 47 | 19416 | 44.6601 | 0.1023 | 0.0098 | 0.0097 |
| GO:0002455~humoral immune response mediated by circulating immunoglobulin | 4 | C3, IGHG3, IGHG2, IGHA2 | 1.22E-04 | 37 | 52 | 19416 | 40.3659 | 0.1360 | 0.0122 | 0.0121 |
| GO:0006956~complement activation | 4 | C3, IGHG3, IGHG2, IGHA2 | 2.26E-04 | 37 | 64 | 19416 | 32.7973 | 0.2378 | 0.0209 | 0.0207 |
| GO:0019730~antimicrobial humoral response | 4 | IGHG3, IGHG2, PRTN3, IGHA2 | 0.0049 | 37 | 186 | 19416 | 11.2851 | 0.9973 | 0.2805 | 0.2779 |
| GO:0002429~immune response-activating cell surface receptor signaling pathway | 4 | C3, IGHG3, IGHG2, IGHA2 | 0.0120 | 37 | 258 | 19416 | 8.1358 | 1.0000 | 0.4806 | 0.4762 |
| GO:0031348~negative regulation of defense response | 4 | APOA1, SFN, IGHA2, YWHAZ | 0.0212 | 37 | 320 | 19416 | 6.5595 | 1.0000 | 0.6499 | 0.6439 |
| GO:0002757~immune response-activating signaling pathway | 4 | C3, IGHG3, IGHG2, IGHA2 | 0.0252 | 37 | 342 | 19416 | 6.1375 | 1.0000 | 0.7048 | 0.6983 |
| GO:0050808~synapse organization | 4 | C3, SPARCL1, PFN1, EZR | 0.0321 | 37 | 376 | 19416 | 5.5825 | 1.0000 | 0.8397 | 0.8320 |
| GO:0002253~activation of immune response | 4 | C3, IGHG3, IGHG2, IGHA2 | 0.0416 | 37 | 417 | 19416 | 5.0336 | 1.0000 | 0.9822 | 0.9733 |

| **Category/Term** | **Count** | **Genes** | ***p*-value** | **List total** | **Pop hits** | **Pop total** | **Fold enrichment** | **Bonferroni** | **Benjamini** | **FDR** |
| --- | --- | --- | --- | --- | --- | --- | --- | --- | --- | --- |
| **Biological process** |  |  |  |  |  |  |  |  |  |  |
| GO:0050853~B cell receptor signaling pathway | 3 | IGHG3, IGHG2, IGHA2 | 0.0057 | 37 | 61 | 19416 | 25.8077 | 0.9990 | 0.3123 | 0.3094 |
| GO:0002526~acute inflammatory response | 3 | FCGR3A, SERPINA1, IGHA2 | 0.0094 | 37 | 79 | 19416 | 19.9275 | 1.0000 | 0.4195 | 0.4157 |
| GO:0019731~antibacterial humoral response | 3 | IGHG3, IGHG2, IGHA2 | 0.0106 | 37 | 84 | 19416 | 18.7413 | 1.0000 | 0.4394 | 0.4354 |
| GO:0045824~negative regulation of innate immune response | 3 | SFN, IGHA2, YWHAZ | 0.0156 | 37 | 103 | 19416 | 15.2842 | 1.0000 | 0.5573 | 0.5522 |
| GO:0050764~regulation of phagocytosis | 3 | C3, APOA1, PRTN3 | 0.0174 | 37 | 109 | 19416 | 14.4428 | 1.0000 | 0.5806 | 0.5753 |
| GO:0002832~negative regulation of response to biotic stimulus | 3 | SFN, IGHA2, YWHAZ | 0.0270 | 37 | 138 | 19416 | 11.4078 | 1.0000 | 0.7215 | 0.7149 |
| GO:0050851~antigen receptor-mediated signaling pathway | 3 | IGHG3, IGHG2, IGHA2 | 0.0379 | 37 | 166 | 19416 | 9.4836 | 1.0000 | 0.9201 | 0.9117 |
| GO:0051345~positive regulation of hydrolase activity | 3 | SERPINB3, APOA1, PRTN3 | 0.0487 | 37 | 191 | 19416 | 8.2423 | 1.0000 | 1.0000 | 0.9917 |
| GO:0002438~acute inflammatory response to antigenic stimulus | 2 | FCGR3A, IGHA2 | 0.0382 | 37 | 21 | 19416 | 49.9768 | 1.0000 | 0.9201 | 0.9117 |
| GO:0034114~regulation of heterotypic cell-cell adhesion | 2 | FGG, APOA1 | 0.0436 | 37 | 24 | 19416 | 43.7297 | 1.0000 | 0.9894 | 0.9803 |
| GO:0030449~regulation of complement activation | 2 | C3, IGHA2 | 0.0436 | 37 | 24 | 19416 | 43.7297 | 1.0000 | 0.9894 | 0.9803 |
| GO:0042026~protein refolding | 2 | HSPA8, HSPA6 | 0.0489 | 37 | 27 | 19416 | 38.8709 | 1.0000 | 1.0000 | 0.9917 |
| GO:0002757~immune response-activating signaling pathway | 4 | C3, IGHG3, IGHG2, IGHA2 | 0.0252 | 37 | 342 | 19416 | 6.1375 | 1.0000 | 0.7048 | 0.6983 |
| GO:0050808~synapse organization | 4 | C3, SPARCL1, PFN1, EZR | 0.0321 | 37 | 376 | 19416 | 5.5825 | 1.0000 | 0.8397 | 0.8320 |
| GO:0002253~activation of immune response | 4 | C3, IGHG3, IGHG2, IGHA2 | 0.0416 | 37 | 417 | 19416 | 5.0336 | 1.0000 | 0.9822 | 0.9733 |
| GO:0050853~B cell receptor signaling pathway | 3 | IGHG3, IGHG2, IGHA2 | 0.0057 | 37 | 61 | 19416 | 25.8077 | 0.9990 | 0.3123 | 0.3094 |
| GO:0002526~acute inflammatory response | 3 | FCGR3A, SERPINA1, IGHA2 | 0.0094 | 37 | 79 | 19416 | 19.9275 | 1.0000 | 0.4195 | 0.4157 |
| GO:0019731~antibacterial humoral response | 3 | IGHG3, IGHG2, IGHA2 | 0.0106 | 37 | 84 | 19416 | 18.7413 | 1.0000 | 0.4394 | 0.4354 |
| GO:0045824~negative regulation of innate immune response | 3 | SFN, IGHA2, YWHAZ | 0.0156 | 37 | 103 | 19416 | 15.2842 | 1.0000 | 0.5573 | 0.5522 |
| GO:0050764~regulation of phagocytosis | 3 | C3, APOA1, PRTN3 | 0.0174 | 37 | 109 | 19416 | 14.4428 | 1.0000 | 0.5806 | 0.5753 |

| **Category/Term** | **Count** | **Genes** | ***p*-value** | **List total** | **Pop hits** | **Pop total** | **Fold enrichment** | **Bonferroni** | **Benjamini** | **FDR** |
| --- | --- | --- | --- | --- | --- | --- | --- | --- | --- | --- |
| **Biological process** |  |  |  |  |  |  |  |  |  |  |
| GO:0002832~negative regulation of response to biotic stimulus | 3 | SFN, IGHA2, YWHAZ | 0.0270 | 37 | 138 | 19416 | 11.4078 | 1.0000 | 0.7215 | 0.7149 |
| GO:0050851~antigen receptor-mediated signaling pathway | 3 | IGHG3, IGHG2, IGHA2 | 0.0379 | 37 | 166 | 19416 | 9.4836 | 1.0000 | 0.9201 | 0.9117 |
| GO:0051345~positive regulation of hydrolase activity | 3 | SERPINB3, APOA1, PRTN3 | 0.0487 | 37 | 191 | 19416 | 8.2423 | 1.0000 | 1.0000 | 0.9917 |
| GO:0002438~acute inflammatory response to antigenic stimulus | 2 | FCGR3A, IGHA2 | 0.0382 | 37 | 21 | 19416 | 49.9768 | 1.0000 | 0.9201 | 0.9117 |
| GO:0034114~regulation of heterotypic cell-cell adhesion | 2 | FGG, APOA1 | 0.0436 | 37 | 24 | 19416 | 43.7297 | 1.0000 | 0.9894 | 0.9803 |
| GO:0030449~regulation of complement activation | 2 | C3, IGHA2 | 0.0436 | 37 | 24 | 19416 | 43.7297 | 1.0000 | 0.9894 | 0.9803 |
| GO:0042026~protein refolding | 2 | HSPA8, HSPA6 | 0.0489 | 37 | 27 | 19416 | 38.8709 | 1.0000 | 1.0000 | 0.9917 |
| **Cellular compartment** |  |  |  |  |  |  |  |  |  |  |
| GO:0005576~extracellular region | 35 | CDA, SERPINA1, IGHV6-1, CRISP3, GDI2, IGHV4-39, C3, IGHG3, NPEPPS, GANAB, FCGR3A, IGHG2, IGLV2-14, IGLV7-46, IGLV1-44, IGLV2-18, SFN, IGHA2, SERPINB3, HSPA8, IGHV3-74, FGG, HSPA6, APOA1, ARPC4, IGKV1D-33, YWHAZ, SERPINB5, IGKV2D-29, IGLL1, IGKV4-1, SPARCL1, PRTN3, EZR, PFN1 | 5.56E-20 | 37 | 4743 | 20747 | 4.1378 | 1.03E-17 | 1.03E-17 | 8.34E-18 |
| GO:0016020~membrane | 32 | SERPINA1, IGHV6-1, CRISP3, GDI2, IGHV4-39, C3, IGHG3, NPEPPS, GANAB, FCGR3A, IGHG2, IGLV2-14, IGLV7-46, IGLV1-44, IGLV2-18, IGHA2, SERPINB3, HSPA8, IGHV3-74, FGG, HSPA6, APOA1, IGKV1D-33, SERPINB5, IGKV2D-29, IGLL1, IGKV4-1, DSG1, PRTN3, ADSS1, EZR, PFN1 | 9.28E-06 | 37 | 1052 | 20747 | 1.7050 | 0.0017 | 1.07E-04 | 8.70E-05 |
| GO:0071944~cell periphery | 28 | SERPINA1, IGHV6-1, CRISP3, IGHV4-39, C3, IGHG3, FCGR3A, IGHG2, IGLV2-14, IGLV7-46, IGLV1-44, IGLV2-18, IGHA2, SERPINB3, HSPA8, IGHV3-74, FGG, HSPA6, APOA1, IGKV1D-33, SERPINB5, IGKV2D-29, IGKV4-1, SPARCL1, DSG1, PRTN3, EZR, PFN1 | 2.06E-07 | 37 | 6739 | 20747 | 2.3298 | 3.80E-05 | 2.72E-06 | 2.20E-06 |

| **Category/Term** | **Count** | **Genes** | ***p*-value** | **List total** | **Pop hits** | **Pop total** | **Fold enrichment** | **Bonferroni** | **Benjamini** | **FDR** |
| --- | --- | --- | --- | --- | --- | --- | --- | --- | --- | --- |
| **Cellular compartment** |  |  |  |  |  |  |  |  |  |  |
| GO:0005615~extracellular space | 26 | SERPINA1, CRISP3, GDI2, C3, IGHG3, NPEPPS, GANAB, FCGR3A, IGHG2, IGLV2-14, SFN, IGHA2, SERPINB3, HSPA8, FGG, HSPA6, APOA1, ARPC4, IGKV1D-33, YWHAZ, SERPINB5, IGKV4-1, SPARCL1, PRTN3, EZR, PFN1 | 7.70E-12 | 37 | 3607 | 20747 | 4.0419 | 1.42E-09 | 1.78E-10 | 1.44E-10 |
| GO:0031982~vesicle | 25 | CDA, SERPINA1, CRISP3, GDI2, C3, IGHG3, NPEPPS, GANAB, FCGR3A, IGHG2, IGLV2-14, SFN, IGHA2, SERPINB3, HSPA8, FGG, HSPA6, APOA1, ARPC4, IGKV1D-33, YWHAZ, DSG1, PRTN3, EZR, PFN1 | 3.28E-09 | 37 | 4296 | 20747 | 3.2631 | 6.06E-07 | 5.05E-08 | 4.09E-08 |
| GO:0005886~plasma membrane | 24 | SERPINB3, HSPA8, IGHV6-1, IGHV3-74, HSPA6, FGG, APOA1, IGHV4-39, IGKV1D-33, SERPINB5, C3, IGHG3, FCGR3A, IGKV2D-29, IGHG2, IGLV2-14, IGLV7-46, IGLV1-44, IGKV4-1, DSG1, IGLV2-18, PRTN3, EZR, IGHA2 | 2.91E-05 | 37 | 6253 | 20747 | 2.1522 | 0.0054 | 2.83E-04 | 2.29E-04 |
| GO:0032991~protein-containing complex | 23 | HSPA8, IGHV6-1, IGHV3-74, HSPA6, FGG, APOA1, IGHV4-39, ARPC4, IGKV1D-33, C3, IGHG3, FCGR3A, IGKV2D-29, GANAB, IGHG2, IGLV2-14, IGLL1, IGLV7-46, IGLV1-44, IGKV4-1, IGLV2-18, EZR, IGHA2 | 2.97E-04 | 37 | 6630 | 20747 | 1.9453 | 0.0535 | 0.0022 | 0.0018 |
| GO:0070062~extracellular exosome | 22 | SERPINB3, HSPA8, SERPINA1, GDI2, HSPA6, FGG, APOA1, ARPC4, IGKV1D-33, YWHAZ, C3, IGHG3, NPEPPS, FCGR3A, GANAB, IGHG2, IGLV2-14, PRTN3, SFN, PFN1, EZR, IGHA2 | 5.18E-12 | 37 | 2242 | 20747 | 5.5023 | 9.59E-10 | 1.78E-10 | 1.44E-10 |
| GO:1903561~extracellular vesicle | 22 | SERPINB3, HSPA8, SERPINA1, GDI2, HSPA6, FGG, APOA1, ARPC4, IGKV1D-33, YWHAZ, C3, IGHG3, NPEPPS, FCGR3A, GANAB, IGHG2, IGLV2-14, PRTN3, SFN, PFN1, EZR, IGHA2 | 1.24E-11 | 37 | 2345 | 20747 | 5.2606 | 2.29E-09 | 2.09E-10 | 1.70E-10 |
| GO:0065010~extracellular membrane-bounded organelle | 22 | SERPINB3, HSPA8, SERPINA1, GDI2, HSPA6, FGG, APOA1, ARPC4, IGKV1D-33, YWHAZ, C3, IGHG3, NPEPPS, FCGR3A, GANAB, IGHG2, IGLV2-14, PRTN3, SFN, PFN1, EZR, IGHA2 | 1.25E-11 | 37 | 2346 | 20747 | 5.2584 | 2.30E-09 | 2.09E-10 | 1.70E-10 |

| **Category/Term** | **Count** | **Genes** | ***p*-value** | **List total** | **Pop hits** | **Pop total** | **Fold enrichment** | **Bonferroni** | **Benjamini** | **FDR** |
| --- | --- | --- | --- | --- | --- | --- | --- | --- | --- | --- |
| **Cellular compartment** |  |  |  |  |  |  |  |  |  |  |
| GO:0043230~extracellular organelle | 22 | SERPINB3, HSPA8, SERPINA1, GDI2, HSPA6, FGG, APOA1, ARPC4, IGKV1D-33, YWHAZ, C3, IGHG3, NPEPPS, FCGR3A, GANAB, IGHG2, IGLV2-14, PRTN3, SFN, PFN1, EZR, IGHA2 | 1.25E-11 | 37 | 2346 | 20747 | 5.2583 | 2.30E-09 | 2.09E-10 | 1.70E-10 |
| GO:0012505~endomembrane system | 17 | SERPINB3, HSPA8, CDA, SERPINA1, CRISP3, GDI2, HSPA6, FGG, APOA1, C3, GANAB, IGLL1, DSG1, SPARCL1, PRTN3, EZR, IGHA2 | 0.0074 | 37 | 5117 | 20747 | 1.8629 | 0.7486 | 0.0382 | 0.0310 |
| GO:0031410~cytoplasmic vesicle | 16 | SERPINB3, HSPA8, CDA, SERPINA1, CRISP3, GDI2, HSPA6, FGG, APOA1, YWHAZ, C3, GANAB, DSG1, PRTN3, EZR, IGHA2 | 1.33E-05 | 37 | 2625 | 20747 | 3.4178 | 0.0025 | 1.43E-04 | 1.16E-04 |
| GO:0097708~intracellular vesicle | 16 | SERPINB3, HSPA8, CDA, SERPINA1, CRISP3, GDI2, HSPA6, FGG, APOA1, YWHAZ, C3, GANAB, DSG1, PRTN3, EZR, IGHA2 | 1.39E-05 | 37 | 2634 | 20747 | 3.4061 | 0.0026 | 1.43E-04 | 1.16E-04 |
| GO:0019814~immunoglobulin complex | 14 | IGHV6-1, IGHV3-74, IGHV4-39, IGKV1D-33, IGHG3, IGKV2D-29, IGHG2, IGLV2-14, IGLL1, IGLV7-46, IGLV1-44, IGKV4-1, IGLV2-18, IGHA2 | 1.80E-17 | 37 | 213 | 20747 | 36.8555 | 3.33E-15 | 1.66E-15 | 1.35E-15 |
| GO:0030141~secretory granule | 13 | SERPINB3, HSPA8, CDA, SERPINA1, CRISP3, GDI2, HSPA6, FGG, APOA1, C3, DSG1, PRTN3, IGHA2 | 2.90E-08 | 37 | 932 | 20747 | 7.8213 | 5.37E-06 | 4.13E-07 | 3.35E-07 |
| GO:0099503~secretory vesicle | 13 | SERPINB3, HSPA8, CDA, SERPINA1, CRISP3, GDI2, HSPA6, FGG, APOA1, C3, DSG1, PRTN3, IGHA2 | 2.25E-07 | 37 | 1124 | 20747 | 6.4853 | 4.16E-05 | 2.77E-06 | 2.25E-06 |
| GO:0072562~blood microparticle | 12 | C3, IGHG3, HSPA8, IGHG2, FGG, HSPA6, IGKV4-1, APOA1, PFN1, IGKV1D-33, IGHA2, YWHAZ | 1.06E-15 | 37 | 151 | 20747 | 44.5613 | 2.05E-13 | 6.56E-14 | 5.32E-14 |
| GO:0034774~secretory granule lumen | 12 | SERPINB3, C3, HSPA8, CDA, SERPINA1, CRISP3, GDI2, FGG, HSPA6, APOA1, PRTN3, IGHA2 | 5.70E-12 | 37 | 329 | 20747 | 20.4521 | 1.05E-09 | 1.78E-10 | 1.44E-10 |
| GO:0060205~cytoplasmic vesicle lumen | 12 | SERPINB3, C3, HSPA8, CDA, SERPINA1, CRISP3, GDI2, FGG, HSPA6, APOA1, PRTN3, IGHA2 | 6.70E-12 | 37 | 334 | 20747 | 20.1460 | 1.24E-09 | 1.78E-10 | 1.44E-10 |
| GO:0031983~vesicle lumen | 12 | SERPINB3, C3, HSPA8, CDA, SERPINA1, CRISP3, GDI2, FGG, HSPA6, APOA1, PRTN3, IGHA2 | 6.92E-12 | 37 | 335 | 20747 | 20.0858 | 1.28E-09 | 1.78E-10 | 1.44E-10 |

| **Category/Term** | **Count** | **Genes** | ***p*-value** | **List total** | **Pop hits** | **Pop total** | **Fold enrichment** | **Bonferroni** | **Benjamini** | **FDR** |
| --- | --- | --- | --- | --- | --- | --- | --- | --- | --- | --- |
| **Cellular compartment** |  |  |  |  |  |  |  |  |  |  |
| GO:0031012~extracellular matrix | 7 | SERPINA1, CRISP3, FGG, SPARCL1, APOA1, PRTN3, IGHA2 | 4.28E-04 | 37 | 576 | 20747 | 6.8144 | 0.0761 | 0.0030 | 0.0024 |
| GO:0030312~external encapsulating structure | 7 | SERPINA1, CRISP3, FGG, SPARCL1, APOA1, PRTN3, IGHA2 | 4.31E-04 | 37 | 577 | 20747 | 6.8026 | 0.0767 | 0.0030 | 0.0024 |
| GO:0005788~endoplasmic reticulum lumen | 6 | C3, GANAB, SERPINA1, FGG, SPARCL1, APOA1 | 2.38E-04 | 37 | 327 | 20747 | 10.2886 | 0.0431 | 0.0020 | 0.0016 |
| GO:0062023~collagen-containing extracellular matrix | 6 | SERPINA1, FGG, SPARCL1, APOA1, PRTN3, IGHA2 | 9.01E-04 | 37 | 438 | 20747 | 7.6812 | 0.1536 | 0.0057 | 0.0047 |
| GO:0070161~anchoring junction | 6 | HSPA8, GDI2, DSG1, PFN1, EZR, YWHAZ | 0.0218 | 37 | 935 | 20747 | 3.5983 | 0.9829 | 0.1006 | 0.0816 |
| GO:0005775~vacuolar lumen | 5 | SERPINB3, C3, HSPA8, GDI2, PRTN3 | 2.60E-04 | 37 | 180 | 20747 | 15.5758 | 0.0469 | 0.0021 | 0.0017 |
| GO:0101002~ficolin-1-rich granule | 5 | HSPA8, CDA, SERPINA1, HSPA6, DSG1 | 3.00E-04 | 37 | 187 | 20747 | 14.9928 | 0.0541 | 0.0022 | 0.0018 |
| GO:0005925~focal adhesion | 5 | HSPA8, GDI2, PFN1, EZR, YWHAZ | 0.0065 | 37 | 433 | 20747 | 6.4749 | 0.7008 | 0.0354 | 0.0287 |
| GO:0030055~cell-substrate junction | 5 | HSPA8, GDI2, PFN1, EZR, YWHAZ | 0.0070 | 37 | 443 | 20747 | 6.3288 | 0.7293 | 0.0372 | 0.0302 |
| GO:0035578~azurophil granule lumen | 4 | SERPINB3, C3, GDI2, PRTN3 | 5.25E-04 | 37 | 91 | 20747 | 24.6475 | 0.0926 | 0.0035 | 0.0028 |
| GO:1904813~ficolin-1-rich granule lumen | 4 | HSPA8, CDA, SERPINA1, HSPA6 | 0.0013 | 37 | 126 | 20747 | 17.8009 | 0.2210 | 0.0083 | 0.0067 |
| GO:0005766~primary lysosome | 4 | SERPINB3, C3, GDI2, PRTN3 | 0.0025 | 37 | 156 | 20747 | 14.3777 | 0.3686 | 0.0143 | 0.0116 |
| GO:0042582~azurophil granule | 4 | SERPINB3, C3, GDI2, PRTN3 | 0.0025 | 37 | 156 | 20747 | 14.3777 | 0.3686 | 0.0143 | 0.0116 |
| GO:0042571~immunoglobulin complex, circulating | 3 | IGHG3, IGHG2, IGHA2 | 2.26E-04 | 37 | 13 | 20747 | 129.3992 | 0.0409 | 0.0020 | 0.0016 |
| GO:0071735~IgG immunoglobulin complex | 3 | IGHG3, IGHG2, IGLL1 | 2.26E-04 | 37 | 13 | 20747 | 129.3992 | 0.0409 | 0.0020 | 0.0016 |
| GO:0031093~platelet alpha granule lumen | 3 | SERPINA1, FGG, IGHA2 | 0.0062 | 37 | 68 | 20747 | 24.7381 | 0.6839 | 0.0348 | 0.0282 |
| GO:0031091~platelet alpha granule | 3 | SERPINA1, FGG, IGHA2 | 0.0111 | 37 | 92 | 20747 | 18.2847 | 0.8734 | 0.0556 | 0.0450 |
| GO:0042470~melanosome | 3 | HSPA8, GANAB, YWHAZ | 0.0164 | 37 | 113 | 20747 | 14.8866 | 0.9532 | 0.0779 | 0.0631 |
| GO:0048770~pigment granule | 3 | HSPA8, GANAB, YWHAZ | 0.0164 | 37 | 113 | 20747 | 14.8866 | 0.9532 | 0.0779 | 0.0631 |
| GO:0070820~tertiary granule | 3 | CDA, CRISP3, DSG1 | 0.0332 | 37 | 165 | 20747 | 10.1951 | 0.9981 | 0.1498 | 0.1214 |

| **Category/Term** | **Count** | **Genes** | ***p*-value** | **List total** | **Pop hits** | **Pop total** | **Fold enrichment** | **Bonferroni** | **Benjamini** | **FDR** |
| --- | --- | --- | --- | --- | --- | --- | --- | --- | --- | --- |
| **Molecular function** |  |  |  |  |  |  |  |  |  |  |
| GO:0019899~enzyme binding | 13 | SERPINB3, HSPA8, SERPINA1, GDI2, HSPA6, APOA1, ARPC4, YWHAZ, PRTN3, SFN, PFN1, EZR, IGHA2 | 1.41E-04 | 34 | 2152 | 19202 | 3.4117 | 0.0291 | 0.0148 | 0.0140 |
| GO:0003823~antigen binding | 11 | IGHG3, IGHV6-1, IGHV3-74, IGHG2, IGLL1, IGLV2-14, IGLV1-44, IGKV4-1, IGHV4-39, IGKV1D-33, IGHA2 | 9.01E-13 | 34 | 200 | 19202 | 31.0621 | 1.89E-10 | 1.89E-10 | 1.79E-10 |
| GO:0098772~molecular function regulator activity | 10 | SERPINB3, C3, HSPA8, SERPINA1, GDI2, APOA1, SFN, PFN1, IGHA2, SERPINB5 | 0.0161 | 34 | 2375 | 19202 | 2.3780 | 0.9668 | 0.1990 | 0.1886 |
| GO:0042802~identical protein binding | 9 | CDA, SERPINA1, FGG, APOA1, SFN, ADSS1, EZR, IGHA2, YWHAZ | 0.0348 | 34 | 2269 | 19202 | 2.2401 | 0.9994 | 0.3324 | 0.3150 |
| GO:0030234~enzyme regulator activity | 8 | SERPINB3, C3, SERPINA1, GDI2, APOA1, SFN, IGHA2, SERPINB5 | 0.0098 | 34 | 1436 | 19202 | 3.1463 | 0.8743 | 0.1474 | 0.1400 |
| GO:0005102~signaling receptor binding | 8 | C3, IGHG3, HSPA8, IGHG2, FGG, APOA1, PRTN3, IGHA2 | 0.01667 | 34 | 1589 | 19202 | 2.8434 | 0.9700 | 0.1990 | 0.1886 |
| GO:0044877~protein-containing complex binding | 7 | HSPA8, FCGR3A, SPARCL1, APOA1, ARPC4, ADSS1, EZR | 0.0249 | 34 | 1340 | 19202 | 2.9503 | 0.9950 | 0.2488 | 0.2358 |
| GO:0004857~enzyme inhibitor activity | 6 | SERPINB3, C3, SERPINA1, SFN, IGHA2, SERPINB5 | 9.45E-04 | 34 | 449 | 19202 | 7.5470 | 0.1801 | 0.0221 | 0.0209 |
| GO:0050839~cell adhesion molecule binding | 6 | HSPA8, FGG, SFN, PFN1, EZR, YWHAZ | 0.0027 | 34 | 572 | 19202 | 5.9241 | 0.4382 | 0.0524 | 0.0496 |
| GO:0140678~molecular function inhibitor activity | 6 | SERPINB3, C3, SERPINA1, SFN, IGHA2, SERPINB5 | 0.0035 | 34 | 606 | 19202 | 5.5917 | 0.5226 | 0.0615 | 0.0583 |
| GO:0004866~endopeptidase inhibitor activity | 5 | SERPINB3, C3, SERPINA1, IGHA2, SERPINB5 | 2.47E-04 | 34 | 180 | 19202 | 15.6879 | 0.0506 | 0.0153 | 0.0145 |
| GO:0030414~peptidase inhibitor activity | 5 | SERPINB3, C3, SERPINA1, IGHA2, SERPINB5 | 2.92E-04 | 34 | 188 | 19202 | 15.0203 | 0.0594 | 0.0153 | 0.0145 |
| GO:0061135~endopeptidase regulator activity | 5 | SERPINB3, C3, SERPINA1, IGHA2, SERPINB5 | 4.28E-04 | 34 | 208 | 19202 | 13.57601 | 0.0860 | 0.0180 | 0.0170 |
| GO:0061134~peptidase regulator activity | 5 | SERPINB3, C3, SERPINA1, IGHA2, SERPINB5 | 8.66E-04 | 34 | 251 | 19202 | 11.2503 | 0.1663 | 0.0221 | 0.0209 |
| GO:0045296~cadherin binding | 5 | HSPA8, SFN, PFN1, EZR, YWHAZ | 0.0025 | 34 | 336 | 19202 | 8.4042 | 0.4119 | 0.0524 | 0.0496 |
| GO:0004867~serine-type endopeptidase inhibitor activity | 4 | SERPINB3, SERPINA1, IGHA2, SERPINB5 | 8.81E-04 | 34 | 110 | 19202 | 20.5369 | 0.1689 | 0.0221 | 0.0209 |
| GO:0003779~actin binding | 4 | ARPC4, ADSS1, PFN1, EZR | 0.0448 | 34 | 464 | 19202 | 4.8687 | 0.9999 | 0.4089 | 0.3875 |
| GO:0034987~immunoglobulin receptor binding | 3 | IGHG3, IGHG2, IGHA2 | 5.34E-04 | 34 | 20 | 19202 | 84.7147 | 0.1061 | 0.0187 | 0.0177 |

| **Category/Term** | **Count** | **Genes** | ***p*-value** | **List total** | **Pop hits** | **Pop total** | **Fold enrichment** | **Bonferroni** | **Benjamini** | **FDR** |
| --- | --- | --- | --- | --- | --- | --- | --- | --- | --- | --- |
| **Molecular function** |  |  |  |  |  |  |  |  |  |  |
| GO:0045309~protein phosphorylated amino acid binding | 3 | SFN, PFN1, YWHAZ | 0.0048 | 34 | 60 | 19202 | 28.2382 | 0.6331 | 0.0769 | 0.0729 |
| GO:0051219~phosphoprotein binding | 3 | SFN, PFN1, YWHAZ | 0.0113 | 34 | 94 | 19202 | 18.0244 | 0.9089 | 0.1588 | 0.1505 |
| GO:0031072~heat shock protein binding | 3 | HSPA8, HSPA6, APOA1 | 0.0225 | 34 | 135 | 19202 | 12.5503 | 0.9915 | 0.2483 | 0.2353 |

**Supplementary Table 7.** **Pathway profiles of 37 genes exclusively identified in the MF group.**

| **Category/Term** | **Count** | **Genes** | ***p*-value** | **List total** | **Pop hits** | **Pop total** | **Fold enrichment** | **Bonferroni** | **Benjamini** | **FDR** |
| --- | --- | --- | --- | --- | --- | --- | --- | --- | --- | --- |
| **KEGG pathway** |  |  |  |  |  |  |  |  |  |  |
| hsa05150:*Staphylococcus aureus* infection | 4 | C3, FCGR3A, FGG, DSG1 | 0.0012 | 19 | 102 | 8534 | 17.6140 | 0.0765 | 0.0795 | 0.0795 |
| hsa05134:Legionellosis | 3 | C3, HSPA8, HSPA6 | 0.0060 | 19 | 56 | 8534 | 24.0620 | 0.3340 | 0.2027 | 0.2027 |
| hsa04610:Complement and coagulation cascades | 3 | C3, SERPINA1, FGG | 0.0144 | 19 | 88 | 8534 | 15.3122 | 0.6229 | 0.3227 | 0.3227 |
| hsa04141:Protein processing in endoplasmic reticulum | 3 | HSPA8, GANAB, HSPA6 | 0.0490 | 19 | 170 | 8534 | 7.9263 | 0.9654 | 0.7258 | 0.7258 |
| **Reactome pathway** |  |  |  |  |  |  |  |  |  |  |
| R-HSA-168256~Immune System | 22 | SERPINB3, HSPA8, CDA, SERPINA1, CRISP3, GDI2, HSPA6, FGG, IGHV4-39, ARPC4, IGKV1D-33, YWHAZ, C3, IGHG3, NPEPPS, FCGR3A, IGHG2, IGLV2-14, IGLV1-44, IGKV4-1, DSG1, PRTN3 | 1.50E-09 | 31 | 2177 | 11494 | 3.7469 | 3.76E-07 | 5.37E-08 | 4.49E-08 |
| R-HSA-168249~Innate Immune System | 20 | SERPINB3, HSPA8, CDA, SERPINA1, CRISP3, GDI2, HSPA6, FGG, IGHV4-39, ARPC4, IGKV1D-33, C3, IGHG3, FCGR3A, IGHG2, IGLV2-14, IGLV1-44, IGKV4-1, DSG1, PRTN3 | 1.64E-12 | 31 | 1150 | 11494 | 6.4482 | 4.11E-10 | 4.11E-10 | 3.44E-10 |
| R-HSA-1643685~Disease | 16 | HSPA8, FGG, APOA1, IGHV4-39, ARPC4, IGKV1D-33, YWHAZ, C3, IGHG3, FCGR3A, GANAB, IGHG2, IGLV2-14, IGLV1-44, IGKV4-1, SFN | 2.72E-05 | 31 | 1926 | 11494 | 3.0802 | 0.0068 | 2.44E-04 | 2.04E-04 |
| R-HSA-5663205~Infectious disease | 14 | HSPA8, IGHV4-39, ARPC4, IGKV1D-33, YWHAZ, C3, IGHG3, FCGR3A, GANAB, IGHG2, IGLV2-14, IGLV1-44, IGKV4-1, SFN | 1.25E-06 | 31 | 1094 | 11494 | 4.7448 | 3.15E-04 | 1.31E-05 | 1.10E-05 |
| R-HSA-109582~Hemostasis | 13 | SERPINA1, FGG, APOA1, IGHV4-39, IGKV1D-33, YWHAZ, IGLV2-14, IGLL1, IGLV1-44, IGKV4-1, PRTN3, PFN1, IGHA2 | 7.30E-08 | 31 | 699 | 11494 | 6.8957 | 1.83E-05 | 8.72E-07 | 7.30E-07 |
| R-HSA-5653656~Vesicle-mediated transport | 13 | HSPA8, SERPINA1, GDI2, APOA1, IGHV4-39, ARPC4, IGKV1D-33, YWHAZ, IGLV2-14, IGLV1-44, IGKV4-1, SFN, IGHA2 | 1.63E-07 | 31 | 752 | 11494 | 6.4097 | 4.09E-05 | 1.86E-06 | 1.56E-06 |
| **Category/Term** | **Count** | **Genes** | ***p*-value** | **List total** | **Pop hits** | **Pop total** | **Fold enrichment** | **Bonferroni** | **Benjamini** | **FDR** |
| **Reactome pathway** |  |  |  |  |  |  |  |  |  |  |
| R-HSA-9824443~Parasitic Infection Pathways | 10 | C3, IGHG3, FCGR3A, IGHG2, IGLV2-14, IGLV1-44, IGKV4-1, IGHV4-39, ARPC4, IGKV1D-33 | 4.54E-09 | 31 | 231 | 11494 | 16.0508 | 1.14E-06 | 9.49E-08 | 7.94E-08 |
| R-HSA-9658195~Leishmania infection | 10 | C3, IGHG3, FCGR3A, IGHG2, IGLV2-14, IGLV1-44, IGKV4-1, IGHV4-39, ARPC4, IGKV1D-33 | 4.54E-09 | 31 | 231 | 11494 | 16.0508 | 1.14E-06 | 9.49E-08 | 7.94E-08 |
| R-HSA-6798695~Neutrophil degranulation | 10 | SERPINB3, C3, HSPA8, CDA, SERPINA1, CRISP3, GDI2, HSPA6, DSG1, PRTN3 | 2.77E-06 | 31 | 490 | 11494 | 7.5668 | 6.96E-04 | 2.79E-05 | 2.33E-05 |
| R-HSA-1280218~Adaptive Immune System | 10 | C3, NPEPPS, FCGR3A, IGLV2-14, IGLV1-44, FGG, IGKV4-1, IGHV4-39, IGKV1D-33, YWHAZ | 2.32E-04 | 31 | 856 | 11494 | 4.3315 | 0.0565 | 0.0016 | 0.0014 |
| R-HSA-9664422~FCGR3A-mediated phagocytosis | 9 | IGHG3, FCGR3A, IGHG2, IGLV2-14, IGLV1-44, IGKV4-1, IGHV4-39, ARPC4, IGKV1D-33 | 7.48E-10 | 31 | 125 | 11494 | 26.6957 | 1.88E-07 | 3.78E-08 | 3.17E-08 |
| R-HSA-9664407~Parasite infection | 9 | IGHG3, FCGR3A, IGHG2, IGLV2-14, IGLV1-44, IGKV4-1, IGHV4-39, ARPC4, IGKV1D-33 | 7.48E-10 | 31 | 125 | 11494 | 26.6957 | 1.88E-07 | 3.78E-08 | 3.17E-08 |
| R-HSA-9664417~Leishmania phagocytosis | 9 | IGHG3, FCGR3A, IGHG2, IGLV2-14, IGLV1-44, IGKV4-1, IGHV4-39, ARPC4, IGKV1D-33 | 7.48E-10 | 31 | 125 | 11494 | 26.6957 | 1.88E-07 | 3.78E-08 | 3.17E-08 |
| R-HSA-2029482~Regulation of actin dynamics for phagocytic cup formation | 9 | IGHG3, FCGR3A, IGHG2, IGLV2-14, IGLV1-44, IGKV4-1, IGHV4-39, ARPC4, IGKV1D-33 | 9.04E-10 | 31 | 128 | 11494 | 26.0701 | 2.27E-07 | 3.78E-08 | 3.17E-08 |
| R-HSA-2029480~Fcgamma receptor (FCGR) dependent phagocytosis | 9 | IGHG3, FCGR3A, IGHG2, IGLV2-14, IGLV1-44, IGKV4-1, IGHV4-39, ARPC4, IGKV1D-33 | 3.56E-09 | 31 | 152 | 11494 | 21.9537 | 8.93E-07 | 8.93E-08 | 7.47E-08 |
| R-HSA-9824446~Viral Infection Pathways | 9 | HSPA8, GANAB, IGLV2-14, IGLV1-44, IGKV4-1, IGHV4-39, SFN, IGKV1D-33, YWHAZ | 0.0016 | 31 | 895 | 11494 | 3.7285 | 0.3365 | 0.0091 | 0.0076 |
| R-HSA-2029481~FCGR activation | 8 | IGHG3, FCGR3A, IGHG2, IGLV2-14, IGLV1-44, IGKV4-1, IGHV4-39, IGKV1D-33 | 9.05E-10 | 31 | 78 | 11494 | 38.0281 | 2.27E-07 | 3.78E-08 | 3.17E-08 |
| R-HSA-166663~Initial triggering of complement | 8 | C3, IGHG3, IGHG2, IGLV2-14, IGLV1-44, IGKV4-1, IGHV4-39, IGKV1D-33 | 2.31E-09 | 31 | 89 | 11494 | 33.3280 | 5.81E-07 | 7.26E-08 | 6.07E-08 |
| R-HSA-2029485~Role of phospholipids in phagocytosis | 8 | IGHG3, FCGR3A, IGHG2, IGLV2-14, IGLV1-44, IGKV4-1, IGHV4-39, IGKV1D-33 | 2.93E-09 | 31 | 92 | 11494 | 32.2412 | 7.35E-07 | 8.16E-08 | 6.83E-08 |
| R-HSA-9664323~FCGR3A-mediated IL10 synthesis | 8 | IGHG3, FCGR3A, IGHG2, IGLV2-14, IGLV1-44, IGKV4-1, IGHV4-39, IGKV1D-33 | 7.94E-09 | 31 | 106 | 11494 | 27.9830 | 1.99E-06 | 1.53E-07 | 1.28E-07 |
| R-HSA-977606~Regulation of Complement cascade | 8 | C3, IGHG3, IGHG2, IGLV2-14, IGLV1-44, IGKV4-1, IGHV4-39, IGKV1D-33 | 1.32E-08 | 31 | 114 | 11494 | 26.0192 | 3.32E-06 | 2.37E-07 | 1.98E-07 |
| R-HSA-166658~Complement cascade | 8 | C3, IGHG3, IGHG2, IGLV2-14, IGLV1-44, IGKV4-1, IGHV4-39, IGKV1D-33 | 2.38E-08 | 31 | 124 | 11494 | 23.9209 | 5.97E-06 | 3.98E-07 | 3.33E-07 |

| **Category/Term** | **Count** | **Genes** | ***p*-value** | **List total** | **Pop hits** | **Pop total** | **Fold enrichment** | **Bonferroni** | **Benjamini** | **FDR** |
| --- | --- | --- | --- | --- | --- | --- | --- | --- | --- | --- |
| **Reactome pathway** |  |  |  |  |  |  |  |  |  |  |
| R-HSA-9662851~Anti-inflammatory response favouring Leishmania parasite infection | 8 | IGHG3, FCGR3A, IGHG2, IGLV2-14, IGLV1-44, IGKV4-1, IGHV4-39, IGKV1D-33 | 7.02E-08 | 31 | 145 | 11494 | 20.4565 | 1.76E-05 | 8.72E-07 | 7.30E-07 |
| R-HSA-9664433~Leishmania parasite growth and survival | 8 | IGHG3, FCGR3A, IGHG2, IGLV2-14, IGLV1-44, IGKV4-1, IGHV4-39, IGKV1D-33 | 7.02E-08 | 31 | 145 | 11494 | 20.4565 | 1.76E-05 | 8.72E-07 | 7.30E-07 |
| R-HSA-9679506~SARS-CoV Infections | 8 | GANAB, IGLV2-14, IGLV1-44, IGKV4-1, IGHV4-39, SFN, IGKV1D-33, YWHAZ | 1.97E-04 | 31 | 484 | 11494 | 6.1285 | 0.0482 | 0.0014 | 0.0012 |
| R-HSA-173623~Classical antibody-mediated complement activation | 7 | IGHG3, IGHG2, IGLV2-14, IGLV1-44, IGKV4-1, IGHV4-39, IGKV1D-33 | 2.58E-08 | 31 | 72 | 11494 | 36.0475 | 6.47E-06 | 4.04E-07 | 3.38E-07 |
| R-HSA-2168880~Scavenging of heme from plasma | 7 | IGLV2-14, IGLV1-44, IGKV4-1, APOA1, IGHV4-39, IGKV1D-33, IGHA2 | 3.88E-08 | 31 | 77 | 11494 | 33.7067 | 9.73E-06 | 5.72E-07 | 4.79E-07 |
| R-HSA-166786~Creation of C4 and C2 activators | 7 | IGHG3, IGHG2, IGLV2-14, IGLV1-44, IGKV4-1, IGHV4-39, IGKV1D-33 | 4.53E-08 | 31 | 79 | 11494 | 32.8534 | 1.14E-05 | 6.31E-07 | 5.28E-07 |
| R-HSA-2173782~Binding and Uptake of Ligands by Scavenger Receptors | 7 | IGLV2-14, IGLV1-44, IGKV4-1, APOA1, IGHV4-39, IGKV1D-33, IGHA2 | 2.80E-07 | 31 | 107 | 11494 | 24.2563 | 7.02E-05 | 3.05E-06 | 2.56E-06 |
| R-HSA-202733~Cell surface interactions at the vascular wall | 7 | IGLL1, IGLV2-14, IGLV1-44, IGKV4-1, IGHV4-39, IGKV1D-33, IGHA2 | 1.11E-05 | 31 | 201 | 11494 | 12.9125 | 0.0028 | 1.07E-04 | 8.97E-05 |
| R-HSA-198933~Immunoregulatory interactions between a Lymphoid and a non-Lymphoid cell | 7 | C3, FCGR3A, IGLV2-14, IGLV1-44, IGKV4-1, IGHV4-39, IGKV1D-33 | 1.72E-05 | 31 | 217 | 11494 | 11.9605 | 0.0043 | 1.60E-04 | 1.34E-04 |
| R-HSA-76002~Platelet activation, signaling and aggregation | 6 | SERPINA1, FGG, APOA1, PFN1, IGHA2, YWHAZ | 5.67E-04 | 31 | 266 | 11494 | 8.3633 | 0.1327 | 0.0034 | 0.0029 |
| R-HSA-199991~Membrane Trafficking | 6 | HSPA8, SERPINA1, GDI2, SFN, ARPC4, YWHAZ | 0.0248 | 31 | 648 | 11494 | 3.4331 | 0.9981 | 0.1294 | 0.1083 |
| R-HSA-5690714~CD22 mediated BCR regulation | 5 | IGLV2-14, IGLV1-44, IGKV4-1, IGHV4-39, IGKV1D-33 | 2.90E-05 | 31 | 69 | 11494 | 26.8677 | 0.0072 | 2.51E-04 | 2.10E-04 |
| R-HSA-2730905~Role of LAT2/NTAL/LAB on calcium mobilization | 5 | IGLV2-14, IGLV1-44, IGKV4-1, IGHV4-39, IGKV1D-33 | 4.70E-05 | 31 | 78 | 11494 | 23.7676 | 0.0117 | 3.94E-04 | 3.29E-04 |
| R-HSA-2871796~FCERI mediated MAPK activation | 5 | IGLV2-14, IGLV1-44, IGKV4-1, IGHV4-39, IGKV1D-33 | 9.77E-05 | 31 | 94 | 11494 | 19.7220 | 0.0242 | 7.91E-04 | 6.62E-04 |

| **Category/Term** | **Count** | **Genes** | ***p*-value** | **List total** | **Pop hits** | **Pop total** | **Fold enrichment** | **Bonferroni** | **Benjamini** | **FDR** |
| --- | --- | --- | --- | --- | --- | --- | --- | --- | --- | --- |
| **Reactome pathway** |  |  |  |  |  |  |  |  |  |  |
| R-HSA-2871809~FCERI mediated Ca+2 mobilization | 5 | IGLV2-14, IGLV1-44, IGKV4-1, IGHV4-39, IGKV1D-33 | 1.06E-04 | 31 | 96 | 11494 | 19.3112 | 0.0263 | 8.06E-04 | 6.75E-04 |
| R-HSA-983695~Antigen activates B Cell Receptor (BCR) leading to generation of second messengers | 5 | IGLV2-14, IGLV1-44, IGKV4-1, IGHV4-39, IGKV1D-33 | 1.06E-04 | 31 | 96 | 11494 | 19.3112 | 0.0263 | 8.06E-04 | 6.75E-04 |
| R-HSA-8957275~Post-translational protein phosphorylation | 5 | C3, SERPINA1, FGG, SPARCL1, APOA1 | 1.80E-04 | 31 | 110 | 11494 | 16.8534 | 0.0441 | 0.0013 | 0.0011 |
| R-HSA-381426~Regulation of Insulin-like Growth Factor (IGF) transport and uptake by Insulin-like Growth Factor Binding Proteins (IGFBPs) | 5 | C3, SERPINA1, FGG, SPARCL1, APOA1 | 3.12E-04 | 31 | 127 | 11494 | 14.5974 | 0.0753 | 0.0021 | 0.0018 |
| R-HSA-114608~Platelet degranulation | 5 | SERPINA1, FGG, APOA1, PFN1, IGHA2 | 3.51E-04 | 31 | 131 | 11494 | 14.1517 | 0.0843 | 0.0023 | 0.0019 |
| R-HSA-2871837~FCERI mediated NF-kB activation | 5 | IGLV2-14, IGLV1-44, IGKV4-1, IGHV4-39, IGKV1D-33 | 3.61E-04 | 31 | 132 | 11494 | 14.0445 | 0.0867 | 0.0023 | 0.0019 |
| R-HSA-76005~Response to elevated platelet cytosolic Ca2+ | 5 | SERPINA1, FGG, APOA1, PFN1, IGHA2 | 4.05E-04 | 31 | 136 | 11494 | 13.6314 | 0.0966 | 0.0025 | 0.0021 |
| R-HSA-983705~Signaling by the B Cell Receptor (BCR) | 5 | IGLV2-14, IGLV1-44, IGKV4-1, IGHV4-39, IGKV1D-33 | 8.20E-04 | 31 | 164 | 11494 | 11.3041 | 0.1860 | 0.0049 | 0.0041 |
| R-HSA-9679191~Potential therapeutics for SARS | 5 | IGLV2-14, IGLV1-44, IGKV4-1, IGHV4-39, IGKV1D-33 | 8.39E-04 | 31 | 165 | 11494 | 11.2356 | 0.1899 | 0.0049 | 0.0041 |
| R-HSA-2454202~Fc epsilon receptor (FCERI) signaling | 5 | IGLV2-14, IGLV1-44, IGKV4-1, IGHV4-39, IGKV1D-33 | 0.0013 | 31 | 184 | 11494 | 10.0754 | 0.2709 | 0.0072 | 0.0060 |
| R-HSA-140877~Formation of Fibrin Clot (Clotting Cascade) | 3 | FGG, PRTN3, IGHA2 | 0.0051 | 31 | 41 | 11494 | 27.1298 | 0.7208 | 0.0277 | 0.0231 |
| R-HSA-8963896~HDL assembly | 2 | APOA1, IGHA2 | 0.0207 | 31 | 8 | 11494 | 92.6935 | 0.9947 | 0.1105 | 0.0925 |
| R-HSA-9755779~SARS-CoV-2 targets host intracellular signalling and regulatory pathways | 2 | SFN, YWHAZ | 0.0309 | 31 | 12 | 11494 | 61.7957 | 0.9996 | 0.1551 | 0.1297 |
| R-HSA-9614399~Regulation of localization of FOXO transcription factors | 2 | SFN, YWHAZ | 0.0309 | 31 | 12 | 11494 | 61.7957 | 0.9996 | 0.1551 | 0.1297 |

| **Category/Term** | **Count** | **Genes** | ***p*-value** | **List total** | **Pop hits** | **Pop total** | **Fold enrichment** | **Bonferroni** | **Benjamini** | **FDR** |
| --- | --- | --- | --- | --- | --- | --- | --- | --- | --- | --- |
| **Reactome pathway** |  |  |  |  |  |  |  |  |  |  |
| R-HSA-75035~Chk1/Chk2(Cds1) mediated inactivation of Cyclin B:Cdk1 complex | 2 | SFN, YWHAZ | 0.0334 | 31 | 13 | 11494 | 57.0422 | 0.9998 | 0.1645 | 0.1376 |
| R-HSA-111447~Activation of BAD and translocation to mitochondria | 2 | SFN, YWHAZ | 0.0385 | 31 | 15 | 11494 | 49.4366 | 0.9999 | 0.1857 | 0.1553 |
| R-HSA-9735871~SARS-CoV-1 targets host intracellular signalling and regulatory pathways | 2 | SFN, YWHAZ | 0.0410 | 31 | 16 | 11494 | 46.3468 | 1.0000 | 0.1941 | 0.1624 |
| **Wikipathway** |  |  |  |  |  |  |  |  |  |  |
| WP2806:Complement system | 4 | C3, FCGR3A, FGG, APOA1 | 8.75E-04 | 19 | 95 | 8832 | 19.5723 | 0.0594 | 0.0613 | 0.0613 |
| WP5115:Network map of SARS CoV 2 signaling | 4 | HSPA8, IGLL1, FGG, APOA1 | 0.0139 | 19 | 254 | 8832 | 7.3203 | 0.6255 | 0.3251 | 0.3251 |
| WP2272:Pathogenic Escherichia coli infection | 3 | ARPC4, EZR, YWHAZ | 0.0055 | 19 | 55 | 8832 | 25.3550 | 0.3186 | 0.1913 | 0.1913 |

**Supplementary Table 8.** **Mean log_2_ fold-change values of 16 proteins exclusively identified in the CF group.**

| **Protein name** | **Gene** | **Mean Value** | **UniProtKB Entry** |
| --- | --- | --- | --- |
| Immunoglobulin lambda constant 6 | IGLC6 | -4.36852 | P0CF74 |
| Calmodulin-like protein 3 | CALML3 | 0.05198 | P27482 |
| Folate receptor gamma | FOLR3 | 0.24641 | P41439 |
| Galectin-7 | LGALS7 | -0.29578 | P47929 |
| Immunoglobulin heavy variable 3-9 | IGHV3-9 | -0.14908 | P01782 |
| Coronin-1A | CORO1A | 0.34792 | P31146 |
| Keratin, type I cytoskeletal 9 | KRT9 | -0.00022 | P35527 |
| Heat shock protein beta-1 | HSPB1 | -2.03715 | P04792 |
| Isoform 2 of Histone H2A.J  (Histone H2A.J) | H2AFJ (H2AJ) | -1.00966 | Q9BTM1-2 (Q9BTM1) |
| Immunoglobulin lambda variable 1-51 | IGLV1-51 | 0.25229 | P01701 |
| Thioredoxin | TXN | -0.23242 | P10599 |
| Nucleolar protein 8 | NOL8 | 0.78800 | Q76FK4 |
| Ubiquitin-like modifier-activating enzyme 1 | UBA1 | -5.32873 | P22314 |
| Isoform 3 of Zinc finger protein 74 | ZNF74 | 0.83018 | Q16587-3 |
| Protein YIF1B | YIF1B | -1.77954 | Q5BJH7 |
| Leukocyte receptor cluster member 9 | LENG9 | 0.86517 | Q96B70 |

**Supplementary Table 9.** **Key biological function of 16 proteins exclusively identified in the CF group.**

| **UniProtKB Entry** | **Gene** | **Protein name** | **Key biological function** |
| --- | --- | --- | --- |
| P0CF74 | IGLC6 | Immunoglobulin lambda constant 6 | Adaptive immune response, immunoglobulin mediated immune response, B cell receptor signaling pathway |
| P27482 | CALML3 | Calmodulin-like protein 3 | Uncategorized biological process function (based on DAVID analysis) |
| P41439 | FOLR3 | Folate receptor gamma | Cell adhesion, fusion of sperm to egg plasma membrane involved in single fertilization, folic acid transport, sperm-egg recognition |
| P47929 | LGALS7 | Galectin-7 | Apoptotic process, heterophilic cell-cell adhesion via plasma membrane cell adhesion molecules |
| P01782 | IGHV3-9 | Immunoglobulin heavy variable 3-9 | Adaptive immune response, immune response, immunoglobulin mediated immune response |
| P31146 | CORO1A | Coronin-1A | Phagolysosome assembly, calcium ion transport, vesicle fusion, phagocytosis, actin filament organization, regulation of cell shape, epithelial cell migration, cell migration, actin cytoskeleton organization, leukocyte chemotaxis, regulation of actin filament polymerization, negative regulation of vesicle fusion, cell-substrate adhesion, regulation of actin cytoskeleton organization, T cell proliferation, T cell homeostasis, natural killer cell degranulation, negative regulation of neuron apoptotic process, innate immune response, homeostasis of number of cells within a tissue, positive chemotaxis, negative regulation of actin nucleation, regulation of release of sequestered calcium ion into cytosol, neuron apoptotic process, early endosome to recycling endosome transport, cellular response to interleukin-4, positive regulation of T cell migration, |
| P35527 | KRT9 | Keratin, type I cytoskeletal 9 | Spermatogenesis, epidermis development, epithelial cell differentiation, skin development, intermediate filament organization |
| P04792 | HSPB1 | Heat shock protein beta-1 | Regulation of protein phosphorylation, regulation of translational initiation, negative regulation of protein kinase activity, response to unfolded protein, response to heat and virus, regulation of autophagy, positive regulation of interleukin-1 beta production, positive regulation of tumor necrosis factor production, intracellular signal transduction, cellular response to vascular endothelial growth factor stimulus, protein refolding, negative regulation of apoptotic process, regulation of canonical NF-kappaB signal transduction, positive regulation of blood vessel endothelial cell migration, chaperone-mediated protein folding, platelet aggregation, anterograde axonal protein transport, negative regulation of oxidative stress-induced intrinsic apoptotic signaling pathway, positive regulation of endothelial cell chemotaxis |
| Q9BTM1-2 (Q9BTM1) | H2AFJ (H2AJ) | Isoform 2 of Histone H2A.J (Histone H2A.J) | Spermatogenesis, cerebral cortex development, heterochromatin formation, cellular response to gamma radiation, cellular senescence |
| P01701 | IGLV1-51 | Immunoglobulin lambda variable 1-51 | Adaptive immune response, immune response |

| **UniProtKB Entry** | **Gene** | **Protein name** | **Key biological function** |
| --- | --- | --- | --- |
| P10599 | TXN | Thioredoxin | Negative regulation of transcription by RNA polymerase II, response to radiation, positive regulation of peptidyl-serine phosphorylation, positive regulation of DNA binding, cell redox homeostasis, negative regulation of protein export from nucleus, positive regulation of phosphatidylinositol 3-kinase/protein kinase B signal transduction, cellular detoxification of hydrogen peroxide, response to nitric oxide, positive regulation of peptidyl-cysteine S-nitrosylation |
| Q76FK4 | NOL8 | Nucleolar protein 8 | rRNA processing, protein localization to nucleolus |
| P22314 | UBA1 | Ubiquitin-like modifier-activating enzyme 1 | Ubiquitin-dependent protein catabolic process, DNA damage response, protein ubiquitination, protein modification by small protein conjugation |
| Q16587-3 | ZNF74 | Isoform 3 of Zinc finger protein 74 | Regulation of DNA-templated transcription, regulation of transcription by RNA polymerase II |
| Q5BJH7 | YIF1B | Protein YIF1B | Protein targeting to membrane, endoplasmic reticulum to Golgi vesicle-mediated transport, protein transport |
| Q96B70 | LENG9 | Leukocyte receptor cluster member 9 | Uncategorized biological process function (based on DAVID analysis) |

**Supplementary Table 10. Gene Ontology profiles of 16 genes exclusively identified in the CF group.**

| **Category/Term** | **Count** | **Genes** | ***p*-value** | **List total** | **Pop hits** | **Pop total** | **Fold enrichment** | **Bonferroni** | **Benjamini** | **FDR** |
| --- | --- | --- | --- | --- | --- | --- | --- | --- | --- | --- |
| **Biological process** |  |  |  |  |  |  |  |  |  |  |
| GO:0007155~cell adhesion | 4 | HSPB1, FOLR3, LGALS7, CORO1A | 0.0257 | 14 | 989 | 19416 | 5.6091 | 1.0000 | 1.0000 | 1.0000 |
| GO:0002449~lymphocyte mediated immunity | 3 | IGLC6, CORO1A, IGHV3-9 | 0.0142 | 14 | 276 | 19416 | 15.0745 | 0.9997 | 1.0000 | 1.0000 |
| GO:0002443~leukocyte mediated immunity | 3 | IGLC6, CORO1A, IGHV3-9 | 0.0202 | 14 | 333 | 19416 | 12.4942 | 1.0000 | 1.0000 | 1.0000 |
| GO:0002252~immune effector process | 3 | IGLC6, CORO1A, IGHV3-9 | 0.0447 | 14 | 512 | 19416 | 8.1261 | 1.0000 | 1.0000 | 1.0000 |
| **Cellular compartment** |  |  |  |  |  |  |  |  |  |  |
| GO:0031982~vesicle | 12 | H2AJ, IGLV1-51, HSPB1, UBA1, CALML3, FOLR3, TXN, LGALS7, CORO1A, IGHV3-9, KRT9, YIF1B | 5.76E-06 | 15 | 4296 | 20747 | 3.8635 | 7.78E-04 | 1.56E-04 | 1.50E-04 |
| GO:0005576~extracellular region | 12 | H2AJ, IGLV1-51, IGLC6, HSPB1, UBA1, CALML3, FOLR3, TXN, LGALS7, CORO1A, IGHV3-9, KRT9 | 1.59E-05 | 15 | 4743 | 20747 | 3.4994 | 0.0021 | 3.07E-04 | 2.96E-04 |
| GO:0005615~extracellular space | 11 | H2AJ, IGLV1-51, IGLC6, HSPB1, UBA1, CALML3, TXN, LGALS7, CORO1A, IGHV3-9, KRT9 | 1.26E-05 | 15 | 3607 | 20747 | 4.2180 | 0.0017 | 2.84E-04 | 2.73E-04 |
| GO:0070062~extracellular exosome | 10 | H2AJ, IGLV1-51, HSPB1, UBA1, CALML3, TXN, LGALS7, CORO1A, IGHV3-9, KRT9 | 2.39E-06 | 15 | 2242 | 20747 | 6.1692 | 3.22E-04 | 1.18E-04 | 1.14E-04 |
| GO:1903561~extracellular vesicle | 10 | H2AJ, IGLV1-51, HSPB1, UBA1, CALML3, TXN, LGALS7, CORO1A, IGHV3-9, KRT9 | 3.49E-06 | 15 | 2345 | 20747 | 5.8982 | 4.71E-04 | 1.18E-04 | 1.14E-04 |
| GO:0043230~extracellular organelle | 10 | H2AJ, IGLV1-51, HSPB1, UBA1, CALML3, TXN, LGALS7, CORO1A, IGHV3-9, KRT9 | 3.50E-06 | 15 | 2346 | 20747 | 5.8957 | 4.73E-04 | 1.18E-04 | 1.14E-04 |
| GO:0065010~extracellular membrane-bounded organelle | 10 | H2AJ, IGLV1-51, HSPB1, UBA1, CALML3, TXN, LGALS7, CORO1A, IGHV3-9, KRT9 | 3.50E-06 | 15 | 2346 | 20747 | 5.8957 | 4.73E-04 | 1.18E-04 | 1.14E-04 |
| GO:0019814~immunoglobulin complex | 3 | IGLV1-51, IGLC6, IGHV3-9 | 0.0088 | 15 | 213 | 20747 | 19.4808 | 0.6968 | 0.1485 | 0.1430 |
| **Molecular function** |  |  |  |  |  |  |  |  |  |  |
| GO:0003723~RNA binding | 6 | ZNF74, HSPB1, NOL8, UBA1, TXN, CORO1A | 0.0180 | 16 | 2092 | 19202 | 3.4420 | 0.8250 | 0.8637 | 0.8637 |
| GO:0003823~antigen binding | 3 | IGLV1-51, IGLC6, IGHV3-9 | 0.0104 | 16 | 200 | 19202 | 18.0019 | 0.6323 | 0.8637 | 0.8637 |

**Supplementary Table 11.** **Pathway profiles of 16 genes exclusively identified in the CF group.**

| **Category/Term** | **Count** | **Genes** | ***p*-value** | **List total** | **Pop hits** | **Pop total** | **Fold enrichment** | **Bonferroni** | **Benjamini** | **FDR** |
| --- | --- | --- | --- | --- | --- | --- | --- | --- | --- | --- |
| **KEGG pathway** |  |  |  |  |  |  |  |  |  |  |
| hsa05012:Parkinson disease | 3 | UBA1, CALML3, TXN | 0.0190 | 8 | 271 | 8534 | 11.8090 | 0.6710 | 1.0000 | 1.0000 |
| **Reactome pathway** |  |  |  |  |  |  |  |  |  |  |
| R-HSA-1643685~Disease | 6 | H2AJ, IGLV1-51, IGLC6, TXN, CORO1A, IGHV3-9 | 0.0250 | 12 | 1926 | 11494 | 2.9839 | 0.9868 | 0.1295 | 0.1090 |
| R-HSA-168256~Immune System | 6 | IGLV1-51, IGLC6, UBA1, FOLR3, TXN, IGHV3-9 | 0.0407 | 12 | 2177 | 11494 | 2.6399 | 0.9992 | 0.2049 | 0.1725 |
| R-HSA-5663205~Infectious disease | 5 | IGLV1-51, IGLC6, TXN, CORO1A, IGHV3-9 | 0.0156 | 12 | 1094 | 11494 | 4.3777 | 0.9321 | 0.0889 | 0.0749 |
| R-HSA-168249~Innate Immune System | 5 | IGLV1-51, IGLC6, FOLR3, TXN, IGHV3-9 | 0.0185 | 12 | 1150 | 11494 | 4.1645 | 0.9590 | 0.0989 | 0.0833 |
| R-HSA-9824443~Parasitic Infection Pathways | 4 | IGLV1-51, IGLC6, TXN, IGHV3-9 | 0.0012 | 12 | 231 | 11494 | 16.5859 | 0.1819 | 0.0476 | 0.0400 |
| R-HSA-9658195~Leishmania infection | 4 | IGLV1-51, IGLC6, TXN, IGHV3-9 | 0.0012 | 12 | 231 | 11494 | 16.5859 | 0.1819 | 0.0476 | 0.0400 |
| R-HSA-1280218~Adaptive Immune System | 4 | IGLV1-51, IGLC6, UBA1, IGHV3-9 | 0.0432 | 12 | 856 | 11494 | 4.4759 | 0.9995 | 0.2113 | 0.1779 |
| R-HSA-5690714~CD22 mediated BCR regulation | 3 | IGLV1-51, IGLC6, IGHV3-9 | 0.0019 | 12 | 69 | 11494 | 41.6449 | 0.2759 | 0.0476 | 0.0400 |
| R-HSA-173623~Classical antibody-mediated complement activation | 3 | IGLV1-51, IGLC6, IGHV3-9 | 0.0021 | 12 | 72 | 11494 | 39.9097 | 0.2962 | 0.0476 | 0.0400 |
| R-HSA-2168880~Scavenging of heme from plasma | 3 | IGLV1-51, IGLC6, IGHV3-9 | 0.0023 | 12 | 77 | 11494 | 37.3182 | 0.3304 | 0.0476 | 0.0400 |
| R-HSA-2029481~FCGR activation | 3 | IGLV1-51, IGLC6, IGHV3-9 | 0.0024 | 12 | 78 | 11494 | 36.8397 | 0.3373 | 0.0476 | 0.0400 |
| R-HSA-2730905~Role of LAT2/NTAL/LAB on calcium mobilization | 3 | IGLV1-51, IGLC6, IGHV3-9 | 0.0024 | 12 | 78 | 11494 | 36.8397 | 0.3373 | 0.0476 | 0.0400 |
| R-HSA-166786~Creation of C4 and C2 activators | 3 | IGLV1-51, IGLC6, IGHV3-9 | 0.0025 | 12 | 79 | 11494 | 36.3734 | 0.3442 | 0.0476 | 0.0400 |
| R-HSA-166663~Initial triggering of complement | 3 | IGLV1-51, IGLC6, IGHV3-9 | 0.0031 | 12 | 89 | 11494 | 32.2865 | 0.4136 | 0.0476 | 0.0400 |
| **Category/Term** | **Count** | **Genes** | ***p*-value** | **List total** | **Pop hits** | **Pop total** | **Fold enrichment** | **Bonferroni** | **Benjamini** | **FDR** |
| **Reactome pathway** |  |  |  |  |  |  |  |  |  |  |
| R-HSA-2871796~FCERI mediated MAPK activation | 3 | IGLV1-51, IGLC6, IGHV3-9 | 0.0035 | 12 | 94 | 11494 | 30.5691 | 0.4480 | 0.0476 | 0.0400 |
| R-HSA-983695~Antigen activates B Cell Receptor (BCR) leading to generation of second messengers | 3 | IGLV1-51, IGLC6, IGHV3-9 | 0.0036 | 12 | 96 | 11494 | 29.9323 | 0.4617 | 0.0476 | 0.0400 |
| R-HSA-2871809~FCERI mediated Ca^+2^ mobilization | 3 | IGLV1-51, IGLC6, IGHV3-9 | 0.0036 | 12 | 96 | 11494 | 29.9323 | 0.4617 | 0.0476 | 0.0400 |
| R-HSA-9664323~FCGR3A-mediated IL10 synthesis | 3 | IGLV1-51, IGLC6, IGHV3-9 | 0.0044 | 12 | 106 | 11494 | 27.1085 | 0.5287 | 0.0510 | 0.0429 |
| R-HSA-2173782~Binding and Uptake of Ligands by Scavenger Receptors | 3 | IGLV1-51, IGLC6, IGHV3-9 | 0.0045 | 12 | 107 | 11494 | 26.8551 | 0.5352 | 0.0510 | 0.0429 |
| R-HSA-977606~Regulation of Complement cascade | 3 | IGLV1-51, IGLC6, IGHV3-9 | 0.0051 | 12 | 114 | 11494 | 25.2061 | 0.5799 | 0.0516 | 0.0435 |
| R-HSA-166658~Complement cascade | 3 | IGLV1-51, IGLC6, IGHV3-9 | 0.0060 | 12 | 124 | 11494 | 23.1734 | 0.6401 | 0.0516 | 0.0435 |
| R-HSA-9664407~Parasite infection | 3 | IGLV1-51, IGLC6, IGHV3-9 | 0.0061 | 12 | 125 | 11494 | 22.9880 | 0.6458 | 0.0516 | 0.0435 |
| R-HSA-9664417~Leishmania phagocytosis | 3 | IGLV1-51, IGLC6, IGHV3-9 | 0.0061 | 12 | 125 | 11494 | 22.9880 | 0.6458 | 0.0516 | 0.0435 |
| R-HSA-9664422~FCGR3A-mediated phagocytosis | 3 | IGLV1-51, IGLC6, IGHV3-9 | 0.0061 | 12 | 125 | 11494 | 22.9880 | 0.6458 | 0.0516 | 0.0435 |
| R-HSA-2029482~Regulation of actin dynamics for phagocytic cup formation | 3 | IGLV1-51, IGLC6, IGHV3-9 | 0.0063 | 12 | 128 | 11494 | 22.4492 | 0.6628 | 0.0516 | 0.0435 |
| R-HSA-2871837~FCERI mediated NF-kB activation | 3 | IGLV1-51, IGLC6, IGHV3-9 | 0.0067 | 12 | 132 | 11494 | 21.7689 | 0.6847 | 0.0523 | 0.0440 |
| R-HSA-9664433~Leishmania parasite growth and survival | 3 | IGLV1-51, IGLC6, IGHV3-9 | 0.0081 | 12 | 145 | 11494 | 19.8172 | 0.7497 | 0.0575 | 0.0484 |
| R-HSA-9662851~Anti-inflammatory response favouring Leishmania parasite infection | 3 | IGLV1-51, IGLC6, IGHV3-9 | 0.0081 | 12 | 145 | 11494 | 19.8172 | 0.7497 | 0.0575 | 0.0484 |
| R-HSA-2029480~Fcgamma receptor (FCGR) dependent phagocytosis | 3 | IGLV1-51, IGLC6, IGHV3-9 | 0.0088 | 12 | 152 | 11494 | 18.9046 | 0.7808 | 0.0604 | 0.0509 |
| **Category/Term** | **Count** | **Genes** | ***p*-value** | **List total** | **Pop hits** | **Pop total** | **Fold enrichment** | **Bonferroni** | **Benjamini** | **FDR** |
| **Reactome pathway** |  |  |  |  |  |  |  |  |  |  |
| R-HSA-983705~Signaling by the B Cell Receptor (BCR) | 3 | IGLV1-51, IGLC6, IGHV3-9 | 0.0102 | 12 | 164 | 11494 | 17.5213 | 0.8276 | 0.0655 | 0.0552 |
| R-HSA-9679191~Potential therapeutics for SARS | 3 | IGLV1-51, IGLC6, IGHV3-9 | 0.0103 | 12 | 165 | 11494 | 17.4152 | 0.8311 | 0.0655 | 0.0552 |
| R-HSA-2454202~Fc epsilon receptor (FCERI) signaling | 3 | IGLV1-51, IGLC6, IGHV3-9 | 0.0127 | 12 | 184 | 11494 | 15.6168 | 0.8885 | 0.0779 | 0.0656 |
| R-HSA-202733~Cell surface interactions at the vascular wall | 3 | IGLV1-51, IGLC6, IGHV3-9 | 0.0151 | 12 | 201 | 11494 | 14.2960 | 0.9257 | 0.0889 | 0.0749 |
| R-HSA-198933~Immunoregulatory interactions between a Lymphoid and a non-Lymphoid cell | 3 | IGLV1-51, IGLC6, IGHV3-9 | 0.0174 | 12 | 217 | 11494 | 13.2419 | 0.9507 | 0.0962 | 0.0810 |
